# Aggressive measures, rising inequalities, and mass formation during the COVID-19 crisis: An overview and proposed way forward

**DOI:** 10.3389/fpubh.2022.950965

**Published:** 2022-08-25

**Authors:** Michaéla C. Schippers, John P. A. Ioannidis, Ari R. Joffe

**Affiliations:** ^1^Department of Technology and Operations Management, Rotterdam School of Management, Erasmus University Rotterdam, Rotterdam, Netherlands; ^2^Department of Medicine, Stanford University, Stanford, CA, United States; ^3^Department of Epidemiology and Population Health, Stanford University, Stanford, CA, United States; ^4^Department of Biomedical Data Science, Stanford University, Stanford, CA, United States; ^5^Department of Statistics, Stanford University, Stanford, CA, United States; ^6^Meta-Research Innovation Center at Stanford (METRICS), Stanford University, Stanford, CA, United States; ^7^Division of Critical Care Medicine, Department of Pediatrics, Stollery Children's Hospital, University of Alberta, Edmonton, AB, Canada; ^8^John Dossetor Health Ethics Center, University of Alberta, Edmonton, AB, Canada

**Keywords:** COVID-19, government response, mass formation, emergency management (EM), rising inequalities

## Abstract

A series of aggressive restrictive measures were adopted around the world in 2020–2022 to attempt to prevent SARS-CoV-2 from spreading. However, it has become increasingly clear the most aggressive (lockdown) response strategies may involve negative side-effects such as a steep increase in poverty, hunger, and inequalities. Several economic, educational, and health repercussions have fallen disproportionately on children, students, young workers, and especially on groups with pre-existing inequalities such as low-income families, ethnic minorities, and women. This has led to a vicious cycle of rising inequalities and health issues. For example, educational and financial security decreased along with rising unemployment and loss of life purpose. Domestic violence surged due to dysfunctional families being forced to spend more time with each other. In the current narrative and scoping review, we describe macro-dynamics that are taking place because of aggressive public health policies and psychological tactics to influence public behavior, such as mass formation and crowd behavior. Coupled with the effect of inequalities, we describe how these factors can interact toward aggravating ripple effects. In light of evidence regarding the health, economic and social costs, that likely far outweigh potential benefits, the authors suggest that, first, where applicable, aggressive lockdown policies should be reversed and their re-adoption in the future should be avoided. If measures are needed, these should be non-disruptive. Second, it is important to assess dispassionately the damage done by aggressive measures and offer ways to alleviate the burden and long-term effects. Third, the structures in place that have led to counterproductive policies should be assessed and ways should be sought to optimize decision-making, such as counteracting groupthink and increasing the level of reflexivity. Finally, a package of scalable positive psychology interventions is suggested to counteract the damage done and improve humanity's prospects.

## Introduction

Historically, health crises have prompted governments and other authorities to act, with differing outcomes [cf. (1–3)]. Global and local health initiatives have long been in place [e.g., see (4)]. For the COVID-19 crisis, governments, and other authorities around the world (e.g., public health agencies, state and county leaders for their citizens, or businesses for their employees) adopted different ways of managing the pandemic. The response often included restrictive population-wide measures, summarized as non-pharmaceutical interventions (NPIs). Many countries opted for long-term strict and aggressive NPIs (5). However, there is little proof that most aggressive measures were more efficient than less disruptive, focused measures [e.g., (6–8)]. Some adopted measures may even have severe negative consequences [for reviews see e.g., (6, 9, 10)]. Furthermore, decision-makers have overly focused on one problem, COVID-19, instead of a more holistic approach (11–13). Together, this crisis management has led to rising inequalities and created new ones (14, 15).

Despite this, many countries opted for long-term strict and aggressive NPIs (5). A recent review and meta-analysis concluded that while lockdowns had little or no beneficial health effects, the economic and social costs were huge (16). Some scientists deem that lockdowns may be the “single biggest public health mistake in history” (17), worrying about long-term repercussions (10, 18). Measures such as closing businesses and disrupting global supply chains (19–21) have taken a toll on the world economy, and on physical and mental health (10, 22, 23). As early as November 2020, the World Bank estimated that the COVID-19 crisis would push 88–115 million people into extreme poverty (24), and a sharp increase in food insecurity worldwide led to hundreds of millions of additional people at risk of starving and food-insecurity (25–28). These macro-economic consequences can worsen mental health issues (29, 30) even cause fragmentation of society (31). Long-term negative economic and health consequences are exacerbated by increasing inequalities (32). Wealth distributions have become more skewed, worsening a pre-pandemic crisis. The top 10% of the global population owns 76% of the total wealth, while the bottom 50% share a mere 2% (33). In September 2021, 1% of the world's population held 45.8% of global wealth (34).

Prior research has shown that, both in the animal kingdom and within the human population, (extreme) levels of inequality often give rise to hierarchies and status dynamics that lead to negative health outcomes (35–39). The Whitehall studies investigating long-term social determinants of health found higher mortality rates in men and women of lower employment grades (40). Up to 20 years of difference in life expectancy has been observed between countries with a large status and economic differences vs. more well-off egalitarian countries (41). Some NPIs may have a large effect on increasing pre-existing inequalities and creating new ones, posing a threat to health and shortening longevity (15). Similarly, certain behavioral interventions along with NPIs used by governments to enforce compliance also worsened inequality. Concurrently, the COVID-19 crisis and the measures taken seem to have offered an opportunity to well-off people who profited from the transformation of life from physical to digital [e.g., (42)], and/or profited from the crisis (43). Many large companies profited, while many small companies crumbled, accelerating pre-existing trends (44).

The rising inequalities have consequences beyond mere financial insecurity, given the dynamism of extreme hierarchical differences (45). From a macro-dynamic perspective, aggressive health policies accompanied by psychological tactics to influence public behavior lead to mass formation and crowd behavior, and the breakdown of normal behavior [cf. (46, 47)]. The burden of financial and food insecurity and deterioration of mental and physical health fall disproportionally on already disadvantaged groups (48, 49), with predictable consequences for social capital and health (50–52). The general insecurity and trauma caused by the insecurity and uncontrollability of the events also contribute to mental health issues (46, 51, 53).

**Figure 1:**
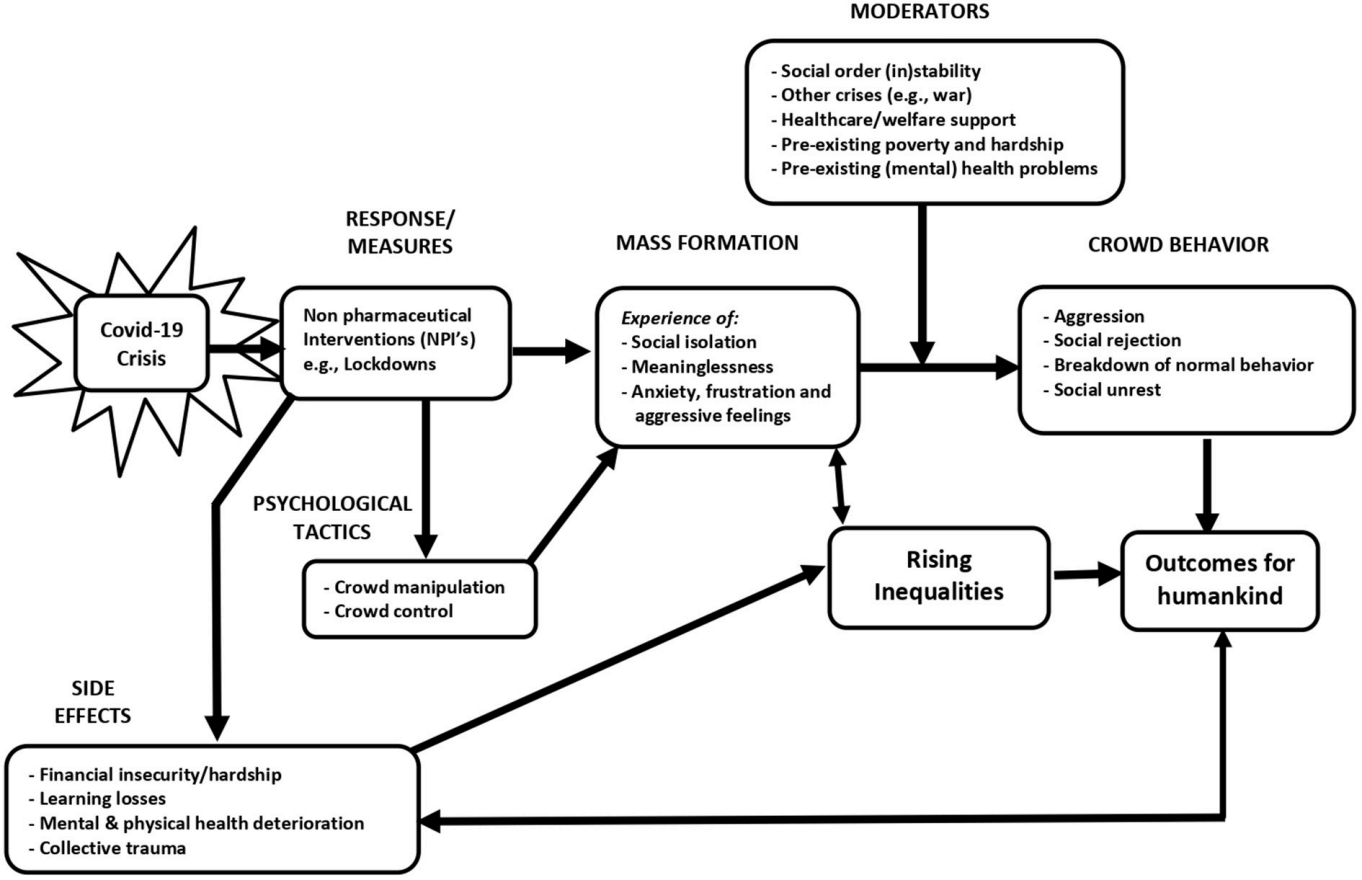
Theoretical model of the consequences of the NPIs on rising inequalities and outcomes for humankind.

The current narrative and scoping review examines the consequences of aggressive NPIs on rising inequalities and adverse outcomes for humankind (see Figure 1). We describe how these NPIs impact mass formation and crowd behavior (Section Aggressive measures, mass formation and crowd behavior), *via* psychological tactics such as crowd manipulation and control (Section Psychological tactics). Section Centralized decision making and one narrative discusses the role of centralized decision making with one narrative and counter movements. Section Collective trauma and conservation of resources addressed issues of collective trauma and offers perspectives from the conservation of resources theory. Section Rising inequalities offers an overview of the resulting increase in inequalities in multiple dimensions: socio-economic, gender, (mental and physical) health, and educational. Section Could we have done better? discusses whether we could have done better, and Section Discussion proposes ways forward. We end with a discussion and recommendations on ways to mitigate the negative effects resulting from aggressive measures.

## Aggressive measures, mass formation, and crowd behavior

During the COVID-19 crisis, governments took the lead in managing the crisis for which they relied on NPIs. However, the 2007 and 2019 reports concluded that high-quality research on NPIs is lacking, and a list of NPIs was assessed in terms of effectiveness (54, 55). In the 2007 paper, it was commented that the scientific base of high quality studies on NPIs is exceedingly small (54), and interventions that were explicitly not recommended were the general use of masks and other protective equipment and social distancing (54). Also, the experts surveyed for this research mentioned that forcibly limiting assembly or movement was legally and ethically problematic; they thought that mandatory long-term community restrictions and compulsory quarantine would lead to public opposition, and practical and logistical problems. It was concluded that voluntary measures and guidelines would be more acceptable and thus effective (54). The 2019 WHO report speaks of spreading cases over a longer period to reduce the height of the peak in “cases” but mentions NPIs such as community use of face masks, border closures, entry- and exit screening, and school closures as generally ineffective. Of the 18 NPIs mentioned in the report, measures such as ventilation and isolation of sick individuals were seen as effective (55). The quality of most studies in the report was rated as (very) low, making it hard to determine effective NPIs, and the possible harmful effects were not weighed. In 2020, a WHO report appeared with considerations on how to ease measures and this report also discussed the importance of human rights protection and the protection of vulnerable populations (56). The extent to which governmental decision-making was flawed is still a matter of debate [e.g., (57)].

Several social psychological theories can explain what could have gone wrong in terms of these interactions. Group processes and crowd psychology predicts that especially in times of crisis people will be inclined to look at governments and authorities to guide their behavior [cf. (1, 3)]. As these authorities respond with guidelines for behavior and NPIs, this can lead to mass formation and crowd formation, similar to the way molecules behave or swarm, with ensuing collective behavior (47, 58, 59). Members of such groups often develop a high degree of emotional like-mindedness, and conventional inhibitions in such groups often decrease (60). In light of the crisis, experts were asked to advise governments, and these used behavioral interventions to steer public behavior in the desired direction and, simultaneously, the debate became highly polarized and politicized (61, 62). Indeed, the behavior of people changed quite radically in the early days of the crisis (63, 64), as psychologists advised governments on how to use psychological tactics to affect behavior change [e.g., (65, 66)]. A special journal issue described the many social group psychological aspects such as impact on societies, social connectedness, and new collective behaviors and inequalities (67). Within the social psychological field of crowd psychology, explanations are offered as to why the behavior of a crowd differs from that of the individuals within the crowd. These theories view the crowd as an entity, where individual responsibility is lost (68). In such a crowd, individuals tend to follow predominant ideas and emotions of the crowd, in a form of shared consciousness, or “collective mind.” Then it becomes relatively easy to violate personal and social norms and such crowds can become destructive (59). This theory may help explain deindividuation and aggression sometimes seen in large groups (69). In such groups, deindividuated people often show more sensitivity and conformance to situation-specific norms and support a social identity model of deindividuation (69).

In the early phase of a crisis, people are inclined to embrace a superordinate level of identity and look for (national) leaders for support and guidance (70). Strong responses toward group members who deviate from new norms are deemed legitimate by many (70, 71), although this may also be dependent on the status of the group member (72), and can change as the crisis progresses. Fluctuations or changes in group behaviors occur later on as people's expectations of a return to normalcy are not met, or if they realize the downsides (70). Indeed, as discontent rises around the globe, citizens may engage in activism (73) and lawsuits against authorities for what they perceived as poor crisis management (74). In times of crisis, blame is often laid on minority groups, who are subsequently scapegoated and persecuted (3). This effect adds to minorities and the poorest already carrying the largest burden for the NPIs (10, 75, 76).

## Psychological tactics

### Crowd manipulation, propaganda, and crowd control

As people turn to leaders in times of crisis (77, 78), leaders have the responsibility to make important and consequential decisions (13). These leaders can choose to intervene in different ways. In general, and especially at the beginning of a crisis, people are inclined to ask for and accept strong leadership [cf. (79, 80)]. Leaders faced the choice between espousing voluntariness in policies or mandating rules and regulations to deal with the crisis (81–83). Although during a crisis leaders tend to enforce rules (84), some voluntariness may be key to trust in government (85). There is some evidence that voluntary measures are more supported than the enforced ones (85), and that voluntariness may offset the experienced disadvantages of policies (5, 83). In general, citizen engagement has many advantages (86). Moreover, it seems that many assumptions on which the NPIs are founded, seem to be biased at best (10, 13, 57). A review of over 100 studies about the COVID-19 crisis handling revealed that overall, the net effects of the policies were negative (87). Studies that suggest substantial benefits of lockdown, typically have flaws or limitations that seriously question the validity, e.g., their counterfactual is based on tenuous assumptions in forecasting models (88), they use interrupted time-series designs without a stable long-term period before and after intervention and without controlling for confounders (89, 90), and/or have no control non-intervention group (i.e., not a difference-in-difference approach) (89, 90), and other flaws (16). Furthermore, it was shown that lockdowns were very costly economically, but probably did not save lives (6, 91). Despite this, citizens generally believed many unfounded COVID-19 scientific claims leading to strong support of NPIs (92). Other options such as involving communities in responses to collective threats, may have avoided many if not all of the negative side effects (63), and voluntary measures may have been better in terms of ethics and human rights (5, 93).

Crowd manipulation, or the use of behavior change techniques based on crowd psychology, could have both intended and unintended consequences (47). While the theory of mass formation has been criticized for being too general (94), it is a meta-theory that seems to be supported by more micro- and middle-range theories on the social psychology of group dynamics and group behavior. These include theories such as group cohesion and intergroup conflict (47). For instance, large increases in perceived threat to a group were significantly related to diminished problem-solving effectiveness (95). A meta-analysis studying 335 effect sizes from 83 samples across 31 countries found that under conditions of strong population norms, norm-behavior associations were also stronger (i.e. people acting according to their norms), and the level of collectivism strengthened these norm effects (96). Governments around the world have strongly communicated a high level of threat and called on norms of collectivism, obedience, and solidarity to excuse NPIs and accompanying harms (10). Overamplifying the harms of COVID-19 leads to citizens becoming more acceptant of the lifestyle changes (97). While these manipulations can in theory benefit the public, the required behaviors have had harmful consequences, especially for vulnerable groups (10, 13, 16, 98). Note that one does not need to invoke some nefarious totalitarianism (99). There can be extreme bonding among people to defeat a real or imagined enemy, in this case, a virus (70). A meta-analysis showed that there is a tendency of ingroup bonding (closing the ranks) combined with a tendency to focus on the outgroup as the source of the threat (100). Even when external threats are not related to a specific outgroup, hostility, prejudice, and discrimination are aimed at outgroups, and detrimental intergroup outcomes occur (1). Dehumanization or the “act of denying outgroup members human-like attributes” [(1), p. 110] may be a mediating factor between a perceived threat and negative behaviors and attitudes toward that group (101). This is strengthened by the moralization of the COVID-19 response which led citizens to believe it is better to impose restrictions than to take no action (102). For the COVID-19 crisis, the superimposed economic crisis contributes to higher levels of hostility and discrimination (and dehumanization) of outgroups to which the cause of the crisis is attributed (1, 103–105). Interestingly, this prejudice against outgroups was not apparent when a system-level explanation for a crisis, i.e. the economic system, was made salient (103). Also, the status of the outgroup moderates this effect: the prejudice is lower when the status of the outgroup is higher (100).

Mass formation concerning reacting to an external threat combined with the resulting extreme inequality can potentially be very harmful [cf. (103, 105)]. Citizen behavior may be unfortunately steered in a direction of societal damage. Mass formation can make people adopt ideas that are incompatible with their previous beliefs. For instance, many people with supposedly progressive ideologies supported harsh measures against unvaccinated people, such as requiring unvaccinated individuals to always remain confined to their homes. Some thought governments should even imprison individuals who publicly questioned vaccine risk-benefit. Moreover, they also thought that unvaccinated individuals should have a tracking device, or be locked up in designated facilities or locations until they are vaccinated (106). These beliefs have nothing to do with improving the uptake of effective vaccines (a most welcome outcome) but delve into other priorities where aggression is the main theme. This kind of dehumanization of a large group could create a whole new kind of inequality: a privileged group of people religiously following governmental response vs. a scapegoated group questioning official policies.

The divide between those groups may have many consequences, from not being willing to work with a co-worker who fails to conform to condoning the violation of basic human rights for such a group with exclusion from society (61). A bias seems to work in the direction of the government responses: a study using a representative sample from 10,270 respondents from 21 countries showed that vaccinated people have a high antipathy against unvaccinated people, 2.5 times more than a more traditional target such as immigrants from the Middle East (61). Interestingly, the antipathy is larger in countries with higher social trust and fewer COVID-19 deaths. In the study, no bias from the unvaccinated toward the vaccinated was detected (61). Why would agreeable and average people hold such beliefs? The answer may be that redirecting the blame toward a scapegoat may help people restore a sense of control, easing feelings of uncertainty (107). For instance, participants “were especially likely to attribute influence over life events to an enemy when the broader social system appeared disordered” [(107); Study 3]. The consequences of crowd behaviors like dehumanization and scapegoating may be quite severe, and it would be advised to work toward reducing intergroup tensions instead of fueling them (1). However, many government responses may have increased these effects rather than reduced them. For political reasons, sometimes governments chose to attribute the blame to some “enemy” while presenting themselves as the savior (3, 108). For the general public, in addition to a social and economic divide, these NPIs and such framing of the message can lead to feelings of social isolation, loss of meaning in life, anxiety, and aggressive feelings (47).

### Experience of social isolation, meaninglessness, anxiety, frustration, and aggressive feelings

The COVID-19 crisis, as with any crisis, spurs feelings of anxiety, frustration, and aggression (109). Social safety theory would predict that social threat greatly impacts human health and behavior (109). Social isolation has led to the experience of meaninglessness, although the role of mindsets about the COVID-19 situation has been important (110). Three mindsets that people formed early in the pandemic, namely considering the pandemic as a catastrophe, as manageable, or as an opportunity, had a self-fulfilling impact on emotions, health behaviors, and well-being (110). In general, the heightened level of mortality salience has been related to heightened frustration and aggression in society [cf. (109)] and especially aggression toward those with opposing world views (111). Human aggression refers to intentional harmful behaviors directed at other individuals, and violence is aggression that has extreme harm as a goal. Hostile aggression is seen as a form of aggression that is rather impulsive or unplanned, while instrumental aggression is premeditated and a proactive form of aggression that is used as a means to an end [for a review see (112)]. Aggressive thoughts and feelings are probably even more common, as many situations and interactions with others can give rise to frustration and aggression. While pre-existing biological and learned tendencies may play a role, the current situation gives rise to a spike in aggressiveness, both verbal (e.g., people blaming certain groups for the current situation and thinking aloud about what should happen to such groups) and actual aggression. There is some evidence that interpersonal aggression and violence increased with aggressive NPIs, especially in places with lockdowns and stay-at-home orders (113, 114). As the crisis continued for much longer than initially expected, aggression and frustration could accumulate, without people having many chances to vent, e.g., by going to the gym.

*Excitation transfer theory* can explain why anger may be extended over longer periods, and this often happens when two or more arousing events are close in terms of time (115). When people are in a survival mode for prolonged periods, they become more fearful, distrustful, irritable, and aggressive (116). Although a survival mode can be an adaptive response to an immediate threat of existential danger, in the long-run over-exposure to stress-response hormones harms mental health and relationships and leads to intergenerational trauma (116, 117). Displaced aggression directed at another person or target, which is not the source of the arousing frustration, can also occur. A meta-analysis showed that the magnitude of the displaced aggression was bigger in a negative setting (e.g., the current crisis). Also, if the provocateur and target were more similar to each other e.g., in terms of gender, race, and/or values, displaced aggression was higher (118).

A study among 2,799 Chinese college students (119) showed that the relationship between fear of COVID-19 and relational online aggressive behavior is mediated by moral disengagement (i.e., the process by which people convince themselves that ethical standards do not apply to them in a certain context, by reframing their behavior as morally acceptable). High mortality salience can also increase aggression, often directed at others who threaten one's worldview (120). Note that terror management can also lead to a more positive way of coping, such as reflecting on the meaning of life (111), and this may be a more effective way of dealing with a crisis (46). However, a study among 1,374 participants in seven Arab countries showed that traumatic stress coupled with collective identity trauma increased death anxiety. This was in turn related to reduced well-being, post-traumatic stress syndrome, anxiety, and depression (45). The authors speak of a vicious cycle of inequalities increasing infection and death from COVID-19 and the COVID-19 crisis increasing inequalities further (45). As many of the behaviors aimed at reducing the spread of the virus, such as hand-washing or masking, can be seen as group rituals (i.e., acts that people regularly repeat together in the same way), symbolizing important group values (e.g., health and safety) people deviating from such rituals provoke anger and moral outrage (10, 121). Individuals more worried about contracting the disease made harsher moral judgments than less worried individuals, even after controlling for political orientation (122). Also, people that were high on health anxiety before the crisis may be more vulnerable to excessive anxiety about COVID-19 (123), and would need therapeutic interventions (124).

There is also evidence that the COVID-19 crisis has increased psychological distress that could be related to proximal and distal defenses against death-related thoughts (45). The crisis has increased anxiety and fear for personal and loved one's physical well-being (125). Conversely, physical activity could act as a buffer (126) but anxiety-buffering outlets such as social networks and sports were inaccessible for many, leaving people vulnerable to experiencing even higher levels of death anxiety (45, 111). A “perfect storm” ensued, whereby stress and anxiety increased and pathways for releasing stress were cut off for many.

Furthermore, all of the social determinants of health were affected; none of these was equally distributed even before the crisis started, but the crisis has accelerated this uneven distribution (127, 128). According to Broadbent and Streicher (129), many of these effects were foreseeable, especially the effects of lockdowns on the Global Poor. During the COVID-19 crisis, commitments to reducing health inequalities were lost from view, or not very salient for wealthy countries, foreseeable health costs were large on deprivation of livelihood, disruption of health services for other conditions, and disruption of education and foreseeable health benefits were minimal (reduction of social contact to the extent modeled was impossible due to overcrowding and non-compliance necessary to sustain a livelihood, the much younger average age while severe COVID affects mostly older people) (129). Much of these effects have been a result of the government's response to the crisis and the choices made in this respect (128). In many countries, decisions were made unilaterally and an official narrative was supported and defended (130).

## Centralized decision making and one narrative

Decision making during a health crisis is difficult as many issues need to be considered concurrently while data may be lacking or massive but still flawed (13, 131). Collective decision-making and intelligence are key to effective decision-making (132). However, sometimes it is falsely assumed that centralized decision making is the only method that may work. Another potential bias may be that a small group of experts is listened to, at the expense of experts that advocate a different route (133). An official narrative approach was followed (130, 134) with counter narratives routinely labeled as misinformation (135). Sometimes the experts in control acquire so much power that they take over even the role of the opposition and dissenters are ostracized (136–138). Authorities have used media and public communication to impose their narrative (134). People and groups challenging the narrative often face dire consequences, from social exclusion to arrest and molestation at demonstrations, in both authoritarian and democratic countries (134). Concurrently, the question has been raised if coercive measures are desirable policy responses, as these have been seen as ineffective and counterproductive in the past (139), leading to distrust in institutions, alienation, and avoidance of care (139–141). The combination of coercive measures and a cancel culture to preserve an official narrative may backfire (139, 142). Public persuasive communication may lead to the opposite effect or behavior than intended (143, 144).

Historically, mixing political ideology with science, when the state regulates science, has led to disastrous outcomes. For instance, a Soviet geneticist favored by Stalin, dominated biology and agricultural science, rejecting Mendelian genetics. The careers and lives of geneticists who opposed him were destroyed, and many were arrested or killed (145, 146). When the Chinese Communists adopted the same approach, starvation killed 30 million people (145). Favoring one ideology at the expense of other views can lead to unwanted outcomes (10, 11, 13, 147), for example, using free speech to shut down free speech (148, 149). The resulting “cancel culture” may frighten other academics who will then be careful in speaking out and/or publishing on certain topics (147). Extremely centralized decision making has other disadvantages, including diminishing democracy, diminished freedoms, and threats to human rights (150–154). Trust in government may diminish, and support for the NPIs may waver (85). While COVID-19 was a major problem, tackling it should never be done to the exclusion of all other problems we face as humanity (57). Decision making should serve most humans, and science can aid here, but it should not be pretended that “science” is perfect and error-free [cf. (155)]. Concurrently, journalism and science should avoid propaganda (154).

### Countermovements

Grassroots movements and countermovements have gained more research attention lately (156–161). As the distribution of power has been unequal throughout history and is typically held by an elite minority, enabling people to use collective power is an important aim of those movements (162). Self-serving (or apparently self-serving) actions of the elite may cause a sharp decrease in trust in institutions for some people, while others keep being trustful. With the COVID-19 crisis, trust in governments and scientific institutions oscillated but mostly decreased (163). People may join countermovements because they give meaning and the opportunity to reinstate dearly held values and beliefs (164). Many citizen activists feel they contribute to a better world in this way; especially the younger generation may be driven more by moral issues rather than political ones (165). However, such groups often face stigmatization and criminalization, undermining of group identity, and institutionalized social subordination (165, 166).

#### The effectiveness of countermovements

In terms of mass formation, possible countermovements have received far less scientific attention (167, 168). Many people may realize that the direction society is moving in does not match with core values, such as humanness (e.g., consideration, empathy), critical thinking, and freedom [cf. (169, 170)]. Indeed, during the COVID-19 crisis, there has been a global wave of social justice movements that draw attention to the negative effects of a multi-dimensional crisis (134). While most of these movements have a strictly non-violent character, the tactics used by these movements range from civil disobedience and (strict) nonviolence to anti-authoritarian strategies and self-defense, and even guerrilla warfare (164). Whether or not these movements are effective and what methods are most effective remains a matter of debate (160). While the authors of this article do not approve of any violence, some writers even argue that violence against a state that has a violence monopoly is sometimes justified and necessary (171). However, recent historical research shows that non-violent approaches are much more effective than violent ones (172). Regardless, the righteousness of such movements can be debated (173). Several authors have claimed that these movements in current times are misinformed and hence see the rise of these movements as dangerous (174). However, simply claiming that those movements are misinformed and labeling all information, not in line with official guidelines as “conspiracy theories” [e.g., (175)] may be too naïve. Some countermovements may be strongly motivated to be well informed. Effectiveness may depend on whether such groups can create space for new social relations, spread awareness, show resilience, have elite support/permission such as that they are shielded from police and military suppression, and are able to improve people's lives (164, 176). A causal relationship between pressure on authorities and change in policies is difficult to determine, but possible (157).

Historical research from 1900 to 2006 comparing the effectiveness of 323 violent vs. non-violent resistance campaigns showed that non-violent civil resistance was more effective in producing change (177). Violent campaigns were successful in 26% of the cases, whereas non-violent campaigns were successful in 50%. In the last 10 years of the research, this effectiveness was reduced to only 6% for violent campaigns vs. 34% for non-violent ones (178–180). Countries in which there were non-violent campaigns were 10 times more likely to transition to democracies within 5 years after those campaigns, than countries with violent campaigns. Interestingly, this was independent of whether the campaign succeeded or failed (178). Effectiveness was bigger under conditions of large, diverse, and sustained participation when the movement was able to elicit loyalty shifts among power elites (e.g., army, police, media, business elites), with campaigns entailing more than protests, with variation in methods used, and when campaigns did not descend into chaos or opt for violent methods despite repression (178). Preparation seems crucial for successful campaigns, for instance in South Africa the anti-apartheid movement organized a boycott of white businesses after preparing for months to become self-sufficient first (181).

The recent decline in the effectiveness of non-violent movements might reflect the smaller size of such campaigns, reliance on more symbolic displays of resistance and mass non-cooperation (such as street demonstrations rather than strikes) that do not weaken the opponent's sources of power, and less disciplined non-violent actions (182). Sometimes even one person can make a difference (183, 184). Della Porta (185) argues that three kinds of ruptures can be brought about by countermovements, often successively: cracking, or sudden ruptures; vibrating, contingently reproducing those ruptures; and sedimenting, stabilization of consequences of the rupture. If these historical lessons apply, perhaps effective countermovements could help in turning around the decisions of implementing non-effective and harmful NPIs, thereby buffering negative long-term effects.

## Collective trauma and conservation of resources

Aggressive measures adversely impact physical and mental health (10, 13, 186). We will focus here on the result of collective trauma or the “psychological reactions to a traumatic event that affects an entire society” [(187), p. 1]. This trauma can affect the collective memory of an entire group and often invokes sense making (188, 189). COVID-19 collective trauma may be large (190). Four mental models seem to be associated with the current collective trauma, namely uncertainty, danger, grotesque, and misery, as well as four primary emotions, namely grief, disgust, anger, and fear (190). Although people have a propensity to hide negative emotions and trauma, the expression of emotions can yield both individual and collective benefits; sharing may alleviate emotional distress and aid in garnering social support (191).

A strong indication of collective hardship is the steep increase in mortality rates among adults under the age of 45, who are largely spared from COVID-19 deaths. Some additional deaths were caused by self-destructive behavior such as substance abuse, homicides, and traffic accidents (98).

Conservation of Resources theory (COR) can serve as an integrative theoretical lens for understanding how people gain and conserve resources (192–194). People differ in the extent to which they are good at gaining tangible resources (e.g., money and property) and intangible resources (e.g., strategic relationships to gain power) (195). According to COR, both individuals and groups, and even societies as a whole strive to obtain and maintain valuable resources (194). There may be an evolutionary need to acquire and conserve resources for survival (194). COR has been used to explain stress outcomes in various contexts, including organizational settings, following traumatic stress and for everyday stressors (192, 196).

**Figure 2:**
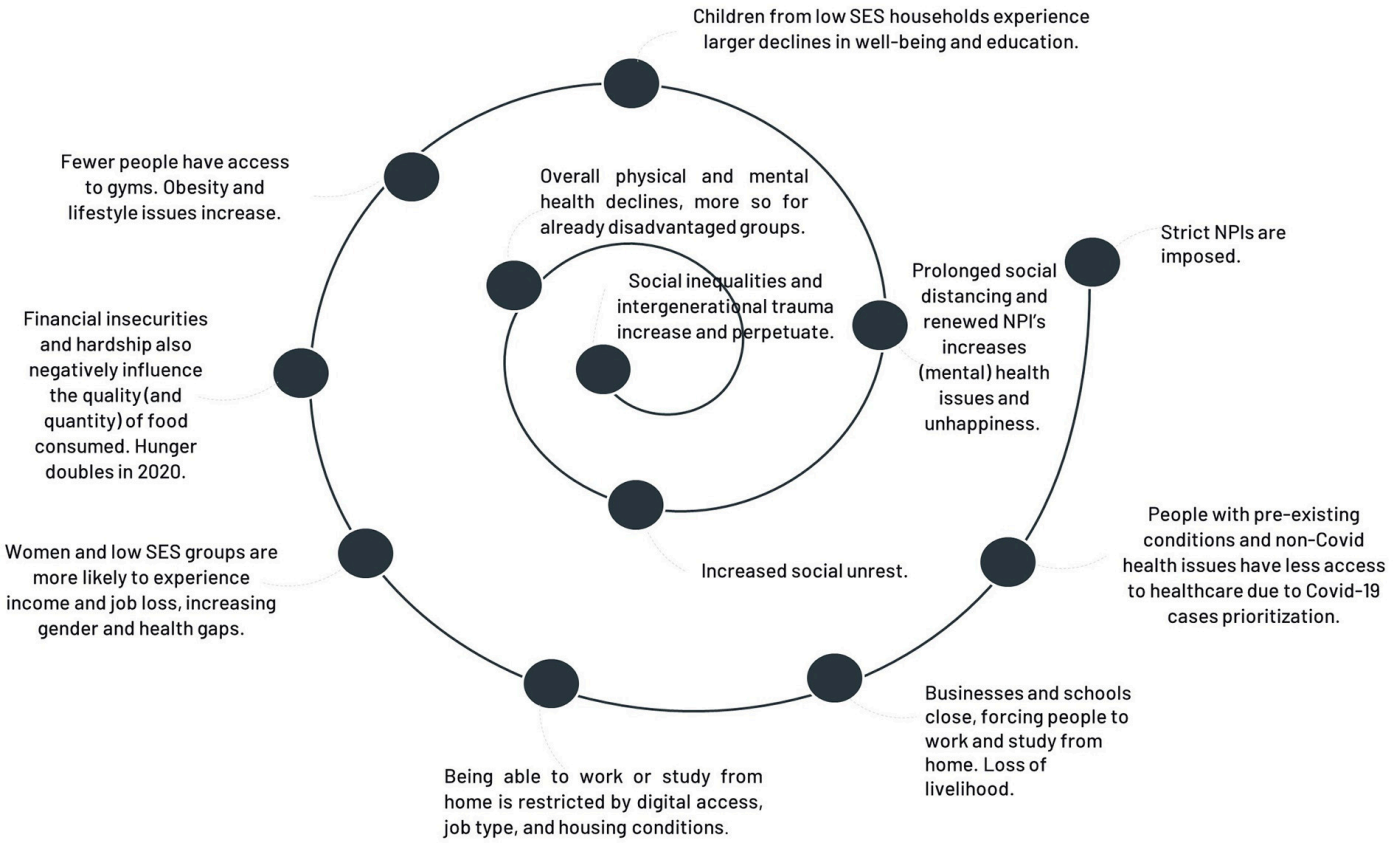
Downward spiral of rising inequalities resulting from aggressive and prolonged NPIs.

Hobfoll speaks of “resource caravan passage ways,” meaning that the ecological conditions often determine the extent to which people can create and sustain resources (194). E.g., women were already on a resource loss before the crisis, but the crisis has exacerbated it, and a resource loss spiral can jeopardize progress toward gender equality (197). For instance, as women work predominantly in service sectors, the shutdown of many such sectors has disproportionately affected them, leading to the largest gender-unemployment gap ever recorded [(198), see also (197)]. This, combined with the increased number of stressors at home, to do more household chores and care tasks, leads to increased stress, less leisure time, and increased burn-out (197). People became more socially conservative during the crisis regarding gender role conformity and gender stereotypes, while political ideology remained constant (199). Stress occurs when resources are lost. In Western contexts, 74 common and important resources are described, including sense of pride, goal accomplishment, hope, personal health, food, help with household chores and childcare, and stable employment (192, 196). The concurrent loss of so many resources during the COVID-19 crisis has been unprecedented [cf. (46), see Figure 2 for a downward spiral in resources].

This can be traumatic for many people, especially given the unpredictability of the duration and intensity of the situation (200). Fear has been identified as a strong predictor of posttraumatic stress disorder, often accompanied by negative thoughts about the self, others, and the world (200). This is compounded by a worldwide sense of insecurity, and loss of personal and social security (201), leading to psychological symptoms of grief (200). Also, job loss has been associated with symptoms of grief and loss of meaning in life (202). Staying-at-home orders are associated with loss of freedom and autonomy as well as loneliness (203), especially when measures were perceived as coercive (204). This may also lead to a fear of coercive policies being enforced over a longer or perhaps indefinite time (139). Fear- and anxiety-related disorders have spiked since 2020 (22). Overall, both tangible and intangible resources were lost during the crisis, thwarting physical and mental health [cf. (200, 205)]. People experiencing extreme resource loss (e.g., losing their income, going through a divorce, losing access to proper health care and ways to cope) may fall prey to the *desperation principle*. This understudied tenet of COR predicts that when people's resources are outstretched or exhausted, they may enter a defensive self-preservation mode in which they behave increasingly aggressive and seemingly irrational (194, 206). They may defensively try to conserve the remaining resources (192). When people are subject to an increased number of stressful events, depression symptoms also increase (207), and major depression is a leading cause of suicide (208). An impact on suicide rates may take years to document. Current research indicates that suicide rates may indeed have increased (186), sometimes after an initial decline in suicides (209). People with more resources before the pandemic may be better suited for resource gain (200) ushering in psychological well-being, health, and functioning (210).

Groups that had fewer resources from the start included minority groups, youngsters, females, and individuals with a mental health history, and economic insecurity (211, 212). Harms induced by NPIs may also be exacerbated by pre-existing or induced lack of stability of the social order in a country or region and in case of pre-existing mental health issues (10, 213). During the crisis, those with pre-existing mental and physical health conditions reported the highest level of emotional distress, although mental health deterioration was population-wide (213). Also, poverty increase in already vulnerable regions made things worse. Additional, extreme events, such as riots and wars may add an extra layer of multiplicative harm (214).

People in comparable circumstances may differ in how resilient they are in dealing with those circumstances (215), and some may experience post-traumatic growth (216). Research by Yi-Feng Chen et al. (217) stresses the role of proactive personality and organizational support in coping with disruptions during COVID-19.

## Rising inequalities

Social inequalities occur when resources within society are distributed unequally, e.g., income, goods, access to information, etc. (218). In the last decades, economic inequality increased in most countries, stabilizing in the 1990s (219), but increasing dramatically since 2020, prompting some authors to refer to this as the “second pandemic” (220). While the focus on making profits has created wealth for large groups of people, resources have become unevenly divided among the total population. There is evidence that economic inequality increased (15). Although this trend was already visible before the crisis started [for a review see (219)], this seems to have accelerated after the start of the crisis (221). While in the last 25 years, 1.1 billion people were lifted from poverty through economic growth (222), during the COVID-19 crisis global extreme poverty rose sharply and in October 2021 it was estimated that 100 million additional people were living in poverty (223). Very early on in the pandemic, warnings were expressed that the negative effects may outweigh possible positive ones (10–12, 57) and ways to optimize decision-making (13) and alternative ways forward were offered (6, 224). Note that other authors disagree and argue that the NPIs are proportional and have substantial benefits [e.g., (225, 226)]. There has indeed been substantial debate on whether lockdowns offer some benefits in reducing at least COVID-19 deaths and many studies have tried to answer this question. In general, these studies have limitations given that no randomized trial has assessed this question and modeling, or observational studies leave substantial uncertainties and are subject to selective reporting and interpretation (227). A meta-analysis has found very small benefits of lockdowns on COVID-19 mortality rates (16), and cost-benefit analyses find that the costs of lockdowns (including what we outline above) far outweigh any potential benefit that may occur (6, 228). Debate and disagreement will likely continue, given that assessments on the relative benefits of lockdown are based largely of weak observational data under very complex circumstances.

Inequalities have several consequences for health, well-being and happiness, and longevity (218, 229). Countries that let inequality increase have lower happiness rates than countries with higher equality (230, 231). Population well-being, consisting of physical, emotional, and social health, explains variation in life expectancy. Communities with high well-being are characterized by engaging in healthy behaviors, strong social connections and support systems (229), and happy people who live longer (232), even though the causal mechanisms can be debated. Several meta-analyses have shown a favorable association between psychological well-being and survival (233), and well-being partially mediates the associations of race, poverty, and education with life expectancy (229). Importantly, life satisfaction and optimism about the future, access to housing, healthcare, and perceptions of safety, were also significantly associated with life expectancy (229). Poor housing conditions were related to greater stress and reduced well-being during the COVID-19 crisis (234). As psychological well-being is affected both directly and indirectly *via* the pandemic and the NPIs (i.e., losing one's job and housing, getting a divorce because of the aforementioned, or because of being quarantined for months), this may lead to more inequalities in terms of income, but also well-being [cf. (46, 235)]. General health and well-being during the crisis have been lowered [for a review see (236)], especially so for vulnerable groups and disadvantaged countries (237, 238). Below we first discuss the various inequalities affected by the pandemic and the adopted NPIs. We should caution that it is often difficult to disentangle how much of these effects were due to the pandemic vs. the measures taken. Occasionally the interaction of the pandemic with the measures taken may have had multiplicative negative effects. Then, we discuss options that may help in breaking this trend. In Table 1, we give a non-exhaustive overview of literature and findings regarding inequalities during the COVID-19 crisis.

**Table 1:** Non-exhaustive overview of the effects on inequality resulting from the non-pharmaceutical interventions enforced in response to the SARS-CoV-2 pandemic.

**Effect on inequalities**	**References**
**Socio-economic status (SES) and ethnic groups**	
Estimates that the side effects of attempting to fully mitigate the COVID-19 pandemic will negatively impact life expectancy. Over 10 years, the negative life expectancy from socio-economic inequalities alone will be around the equivalent of six unmitigated COVID-19 pandemics. This is not considering the negative effects on life expectancy due to increased mental health problems, suicides, and drug abuse	(239)
The effect of the COVID-19 pandemic and lockdowns differed across SES groups, e.g., groups or counties with lower SES had higher infection incidence and mortality	(32, 42, 240, 241)
Racial minorities (Black, Indigenous, and Hispanic) were more at risk of getting infected and had worse COVID-19 health outcomes during the pandemic. Existing inequalities were exacerbated	(42, 128, 242–248)
Children with low SES experienced worse health outcomes during the pandemic due to increased exposure to adverse health determinants (e.g., tobacco, unsuitable food, changes in physical activity, spending more time in front of the screen, less social contact, and more noise	(242, 245, 249–255)
People living in areas with higher levels of pre-existing inequalities experienced more adverse effects during the pandemic	(32, 240, 241, 244, 246, 255–259)
Healthy behaviors (e.g., physical activity, healthy eating) were lower, especially for low SES families	(241, 260)
Geographical economic effects of the crisis. Uneven economic effects uncorrelated to the epidemiological pattern. Lower educational levels related to higher mortality for working-aged women and people between 65 and 79 years old during the crisis. The rise in social inequality because of the burden of the disease and the measures have fallen disproportionally on already disadvantaged groups challenges solidarity and social justice	(32, 240, 246, 255–259, 261, 262)
The pre-existing inequalities of refugee teenagers compounded due to the response to the pandemic, with worse (mental) health outcomes, due to severe economic and service disruptions, as well as low social connectedness	(263)
Ethnic minorities had a lower COVID-19 vaccine uptake, higher mortality rates and larger decreases in life expectancy	(248, 264)
Food insecurities arise for low SES groups due to the rise in poverty, unemployment and food prices. In addition to the economic barriers, people living in rural areas also experienced insecurities due to decreased psychical access to food	(265–268)
Food insecurities lead to an increase in unhealthy eating behaviors (e.g., consuming high caloric products)	(260)
Digital inequalities led to disparate possibilities during the pandemic such as access to COVID-19 vaccinations, the ability to work or study from home and to maintain social connections with friends and family	(258, 269–273)
**Gender inequalities**	
Women experienced higher rates of mental health issues and psychological deterioration than men	(260, 274–277)
Women experienced a higher increase in suicide rates than men	(278, 279)
Women also more often experienced job loss and/or loss of income than men	(247, 276, 277, 280–283)
Gender gaps and unequal distribution of household chores increased during the pandemic. Women reported increased household chores and childcare and decreased leisure time. The propensity to work from home did not differ across genders. In Spain, by May 2020, women from middle-income households with kids experienced 3% larger income loss than men	(274, 277, 280, 283, 284)
Reinforcement of existing gender inequality in academic work. Women were underrepresented as (senior) authors of academic papers during the pandemic, deepening pre-existing inequality. While the quantity of women authored publications seemed to have been on par, quality seemed lower	(285–287)
Women were more exposed to the COVID-19 virus than men due to representing most frontline workers. In Spain, the cumulative incidence rate was higher for women than men	(244, 251, 288)
Males experienced higher COVID-19 mortality rates than females	(242, 244)
The COVID-19 pandemic caused serious setbacks in advancements in solving problems such as child marriages, gender-based violence, and female genital mutilation. Estimates show that 6 months of lockdown led to an additional two million more cases of female genital mutilation, 31 million cases of gender-based violence, and 13 million more child marriages over the next 10 years that wouldn't have occurred otherwise	(289)
**Age group inequalities**	
The risks of mortality from COVID-19 for people aged 60 and above are significantly higher than for younger people. This led to a life expectancy decrease in 27 out of 29 countries included in the study	(245, 251, 290):
Children subjected to school closure and other lockdown measures reported adverse mental health symptoms	(291)
**Health inequalities**	
Patients with non-COVID 19 conditions had less access to treatment and preventive measures during the crisis Taken together with	(244, 292)
other trends, such as privatization of healthcare, already marginalized sections of society were hit harder, leading to worsening existing and creating new health inequalities	
Physical activity health inequality was increased due to differences in access and availability to engage in physical activities during lockdowns	(293)
The switch to remote consultations especially impacted older people, unemployed, people with low SESs, migrants, and men, as these groups were less likely to use remote consultation	(250)
People with pre-existing health conditions (e.g., obesity or malnutrition) had worse COVID-19 outcomes. Oftentimes these people also experienced social inequalities and nutritional disparities long before the crisis	(262, 294–296)
**Mental health inequalities**	
The crisis increased existing mental health conditions and exacerbated preexisting inequalities in that respect. Financial insecurity mediated some of the effect of SES and mental health outcomes. People with a (family) history of mental health disorder also experienced greater difficulties adjusting after lockdown release. SES inequalities in social network, loneliness and mental health increased. A study in Japan showed positive effect on subjective well-being for socially advantaged people vs. negative effects for socially disadvantaged people, widening the gap	(241, 260, 262, 296–302)
**Economic inequalities**	
Income inequality was mainly created by the policy response to the crisis rather than its health consequences. By early June 2020, the pandemic has generated at least 68 million additional poverty years in 150 countries, mainly among already disadvantaged groups. Additionally, the health consequences worsen income inequality	(303)
Working from home increased inequalities in the labor market based on SES, digital access, job type, sector, and hierarchical position. Male, older, highly educated, and highly paid employees benefited from working from home	(42, 244, 257, 260, 273, 283, 304, 305)
Aggressive NPIs increased income inequality and poverty, with vulnerable groups impacted more. In Spain, by May 2020, households in the richest quintile lost about 7% of their income, while the poorest quintile lost 27% of their income	(247, 262, 306–308)
The pandemic did not affect between-country inequality, which continued to decrease as in the previous years	(309)
**Educational inequalities**	
Educational inequalities emerged or increased in terms of parental income, education, internet access, English and technology skills, and/or previous school performance. Search for online learning resources was substantially larger for areas with higher income, better internet access and fewer rural schools in the US. In Germany, daily learning time was halved, from 7.4 h. This decrease was significantly larger for low achievers, who displaced learning time with TV or computer games. In the Netherlands, where access to internet is better than other countries, with a relatively short school closures of 12 weeks, education learning loss sharply increased for students from disadvantaged households	(269–271, 310–312)

### Vulnerable populations

Many authorities responding to the pandemic often stated they aimed to protect the vulnerable. However, several adopted measures seem to have especially hurt this group instead of helping. Several measures disrupted and contracted the social networks of older adults during the crisis. Pre-pandemic racial/ethnic network disparities were exacerbated, with negative consequences for the physical and mental health outcomes of these groups (211). As networks are important not only in daily life, but especially in times of crisis, social distancing led to a limited ability to weather the crisis, especially for vulnerable populations (211). Many countries have chosen to put vulnerable elderly people in complete isolation. This forced social and physical isolation is a serious stressor (313). Resilience may have been further compromised (314, 315), creating paradoxical effects (10). Both regular and routine health care for non-COVID-19 disease was disrupted, posing a threat to health outcomes for many diseases (243, 292). The long-term consequences of the relative neglect of the public health care system, and that people were hesitant to visit their physician for the non-COVID-19 problems (279, 316–319), remain unfathomed. E.g., it was estimated originally that about 28.5 million operations worldwide were postponed during the initial 12-week peak of the crisis (320). Once more, vulnerable populations were hit hardest, increasing pre-existing inequalities (321).

### Economic inequality: The rich got richer and the poor poorer

Economic inequality has hugely increased exacerbating pre-existing inequalities and this seems a self-reinforcing process as lockdown measures continue or keep being imposed (15, 49, 322–324). Hundreds of millions of people were driven into poverty, while others, individuals and corporations, gained (325). This has led to the paradoxical situation that in some countries people were more worried about starvation than becoming ill from COVID-19 (49). Almost 4 billion people, half of the world population, live on <6.70 dollars a day. A review across four continents showed that restrictive NPIs are especially hard on the poor as they unevenly impact the livelihood and socio-economic activities of those groups (326). A World Bank report concluded: “Taken together, COVID-19 has directly offset the reduction in the [poverty] gap between countries observed from 2013 to 2017” (324). Income loss was steepest for the poorest 20% of the world, resulting in the largest impact of the COVID-19 crisis on the world's poorest, increasing the global poverty rate from 7.8 to 9.1 percent by the end of 2021 (327). The effects on inequality and social mobility are expected to be long-term: people who lost income due to the pandemic have been about twice as likely to spend down on assets or savings. Hence, they will be less able to cope with continued or reoccurring income loss. Also, 57% of the people who lost income due to the pandemic have been more likely to go a full day without eating, and the aggregate loss of between 0.3 and 0.9 years of schooling also impacted the poorer families and their economic prospects. Government interventions such as unemployment insurance and benefits for furloughed workers in the short term at least, partially mitigate the effect of the loss of livelihood (14). In Spain, it has been estimated that without those interventions, inequality would have increased by almost 30% in just 1 month (14, 223). However, young people and foreign-born workers profit less from those interventions and experience a large loss of purpose in life (46, 328, 329).

### Educational inequalities

Early in the pandemic, school closures were widespread. In March 2020 schools closed in 138 countries, affecting 80% of students worldwide (214). This is despite a heated scientific debate regarding the effectiveness of school closures on virus transmission. Without a clear answer on the effectiveness of school closures, students' education suffered and the “hurt can last a lifetime” [(330); for a review see (10, 214)]. As early as April 2020 it was stated that school closures would affect poorer children most, as closures also exacerbated food insecurity and the non-school factors (e.g., parental availability for help and supervision, internet access and technology availability, quiet spaces, etc.) that are the primary source of inequalities in educational outcomes (214). Even though many schools switched to online education, this did not help much as a substitute. A study in the Netherlands among 350,000 students showed that students made little or no progress during the school closure and learning loss was “most pronounced among students from disadvantaged homes” [(331), p. 1]. This was despite that the Netherlands was seen as a best-case scenario, with a relatively short lockdown, equitable school funding, and one of the best rates in terms of broad-band access. While for children from high-income families learning might be possible at least theoretically, children from lower income families are faced with numerous hurdles. Besides this, as many parents lost their jobs, these children may be exposed to this stress as well. As “previous recessions have exacerbated levels of child poverty with long-lasting consequences for children's health, well-being, and learning outcomes.” [(214), p. 243], the long-lasting consequences should not be underestimated (332). Recent studies showed a sharp increase in inequalities regarding education (269, 331) and student well-being (333). In addition, homeschooling caused high levels of parental stress (334). Taken together, educational inequalities increased sharply, and student, as well as parent well-being was at stake during and after the school closures.

### Gender inequalities

While the year 2020 was earmarked for reflection on gender inequalities, it has been the year that saw an increase in both existing and new gender inequalities (278). The rising gender inequalities are in the domains of health and well-being, home, domestic violence, work and poverty, and leadership (278). Women reported greater stress and anxiety during lockdowns (335), especially women with children (336), and female students (333). The health and well-being of women were also disproportionally affected, lowering life expectancy, and increasing suicide rates (337). Moreover, reports of abuse, self-harm, and thoughts of suicide/self-harm were higher among women (338). Women were more likely to experience (physical) aggressive interactions in their dream content (339). Also, women's physical and reproductive health was jeopardized, as many countries reallocated medical care toward COVID-19 patients (340). Gender-based violence increased at an alarming rate [for a review see (341)]. Anxiety and depression tripled for pregnant and postpartum women (342). Mothers were more likely to take on more household chores during the crisis and they were responsible for homeschooling (343), and worked on average 5% less, while men worked on average the same number of hours (344). Women with young children reduced their work hours four to five times more than fathers (344).

In academia, pre-existing inequalities persisted, and new ones arose. While academic gender inequalities were already discussed for quite some time [e.g., (345)], the crisis increased pre-existing gender inequalities (346). For instance, in terms of academic output, while men working mainly from home became more productive in the first 10 weeks of the lockdown, and overall research productivity in the US increased by 35%, female productivity dropped by 13%. This productivity gap was found in six more countries (347). While women already faced inequity in terms of having a higher teaching load and more service tasks, which are rewarded less than academic publishing, this was exacerbated when teaching and mentoring had to be done online (347). This is compounded by women having to take on most household tasks, homeschooling, childcare and sometimes caring for aging parents and extended family (343, 348). Also, it was predicted that women's poverty rate would rise by 10% globally as a result of the NPIs, as many service jobs were affected (349). Taken together, women experienced more mental health problems, domestic violence, and a larger burden of household and professional tasks.

### Results of inequalities: Increase in stress

The result of rising inequalities may be an increase in stress and resulting in mental health problems (350). A meta-analysis indeed showed that income inequality was negatively related to mental health (351). In general, humans cause stress on people lower in the hierarchy, and in the last few decades, a lot of research investigated the causes and consequences of this [for a review see (352, 353)]. For instance, Sapolsky researched the question of why primates (including humans) cause each other so much stress. Apes and other primates have more stress-related diseases than any other species, and this seems to be because having spare time in these species is used to cause stress to others, usually lower in the hierarchy (36). Stress levels for low-status baboons were significantly reduced when baboons high in the hierarchy were inadvertently killed due to eating tainted meat (37). The extent to which these studies have validity for human society is debatable. For obvious ethical reasons, it is very difficult to do a study in which extreme hierarchical differences are created and subsequently lifted to study the effects. However, the Whitehall studies, stretching over decades show that status differences and inequalities are related to ill health and mortality, even when controlling for lifestyle (38), and these differences in health outcomes and mortality even stretched until after retirement (352). Interestingly, this was the case even though mental health for low status workers, working in stressful jobs with little autonomy, increased after retirement (354). It goes without question that it is imperative to minimize inequalities.

### Reducing inequalities

Good governance, or the actions governments and organizations take to govern society through laws, norms, power or language, is key to reducing inequalities in society (355). Reducing gender inequalities in academia is also important and several policies are promising (356). An Oxfam report suggested responding to the crisis with several measures to increase equality (357). In general, community development seems to be a promising avenue in this respect (358). Coordination and integration of the health sector and community development may help streamline efforts to influence health and well-being of especially vulnerable groups (358). Evidence-based policy making may help reduce inequalities (359) and to buffer the negative effects of the crisis. Going forward, citizens and governments should act to create a more equal and sustainable world (325). Below, we describe what governments could have done better and what can be learned from this crisis. This examination should not be construed as an effort to blame anyone–a blame culture would be a perpetuation of the crisis and the toxic environment that we described above that fosters inequalities. Conversely, it is important to learn from our mistakes to correct them and not repeat them, close the circle of the pandemic, and be prepared for future pandemics without disrupting life (360).

## Could we have done better?

We could have done better in our response to COVID-19. Vast power was given to experts who had (or claimed) expertise on COVID-19. This resulted in an exclusive focus on illness and deaths from COVID-19, with implemented and mandated NPIs of unprecedented severity, and which had been recommended against in previous pandemic plans (54, 55, 141, 361). These NPIs were also implemented without adequate consideration of their collateral effects (as discussed above and predicted in previous pandemic plans). The response bypassed the lessons learned from past pandemics and other emergencies.

Emergency management (EM) is the prevention and mitigation of, preparedness for, response to, and recovery from emergencies, regardless of the risk/hazard (362). An EM Agency (EMA) is a coordinating agency that coordinates requests from the Subject Matter Agency (the agency dealing with the direct effects of the hazard, here, public health for the COVID-19 hazard), while also dealing with the indirect effects of the hazard (here, pandemic and response) (363). The EMA coordinates the four simultaneous EM critical functions (**Table 3**) during a public emergency, like COVID-19, with direct and indirect effects of the virus and any response to the virus on all of society.

The EM process is the same for any public emergency, including a pandemic. By following the process, the EMA, unlike the public health medical experts, is specifically trained to optimize the response. The seven EM process steps that must occur in any public emergency, and how these should have been taken for this pandemic, are shown in Table 2 (6, 363). By not following the established EM process, the wrong aim, governance, mission analysis, and courses open were more likely to be selected without any published pandemic plan (363). Many negative consequences and exacerbations of inequality discussed above were predictable and should have been considered in risk-benefit analyses (6, 11, 54, 55, 141, 361). Others concluded that crucial parts of the EM process were missed during the pandemic response, although these authors did not recognize that these were components of the EM process and that they were, so to speak, reinventing the wheel (11, 13, 365). In Table 3 we mention some priorities we believe the EM process would have discovered to enable a response with far less collateral damage, and some current priorities necessary for recovery.

**Table 2:** The emergency management process: seven steps and how they should have been applied during the SARS-CoV-2 pandemic.

**Steps in the EM process**	**Specifics of this step during the SARS-CoV-2 pandemic**
1. Identification of the hazard	The hazard is SARS-CoV-2
2. Selection and maintenance of the aim	The aim is to minimize the impact of SARS-CoV-2 and our response on the society of the jurisdiction The aim was not necessarily “to flatten the curve” or “to protect the medical system,” which may be included in objectives
3. Establish a Governance Task Force, to provide leadership for all policy, programs, and actions taken, with many diverse stakeholders involved, and led by the most senior government official (e.g., the provincial premier in the provinces of Canada)	Governance Task Force was not assembled, and public health officers and medical advisors had undue influence
4. Risk/Hazard assessment	The risk from SARS-CoV-2 was very early on known to be extremely age-dependent (especially in older adults with comorbidities), and the potential impacts on critical infrastructure (including healthcare) predictable
5. Mission analysis to determine the *objectives* of *what* needs to be done	For SARS-CoV-2 this includes tasks given (pre-written pandemic response plans) and tasks implied required to meet the aim. This included maintaining confidence in government (by diminishing fear, ensuring mutual aid, and ensuring constant communications), protecting seniors, and protecting critical infrastructure and essential services (e.g., new medical surge capacity, full continued education, continuity of business and economy)
6. Defining courses open/options to determine *how* the mission analysis objectives can be met	This entails determining courses open for each grouping of tasks, as determined by assigned teams with appropriate diverse expertise (to prevent groupthink). Each course open has a full assessment of cost-benefit to justify options, and plan for solutions to expected collateral damage
7. Public issuing of a written comprehensive evidence-based Response Plan	Issuing a written Pandemic Response Plan forms the basis of confidence in government by transparently demonstrably justified due diligence

*References: Joffe and Redman (6), Redman (363), and Redman (364)*.

**Table 3:** Examples of emergency management function priorities in addressing the SARS-CoV-2 pandemic.

**EM function**	**Priorities at the start of the pandemic**	**Priorities mid-2022 for endemic SARS-CoV-2**
Preparation	Define the mission: to ensure minimum impact of SARS-CoV-2 on society as a whole Establish a Governance Task Force as the single decision-making body for policy, programs, and actions, with broad diverse representation, led by the Premier, and coordinated and supported by the Emergency Management Agency Release a comprehensive written Pandemic Response Plan	Define the mission: to ensure minimum impact of endemic SARS-CoV-2 on society as a whole, *and* to recover from the lockdown-based response collateral damage Establish the appropriate Governance Task Force and disband other advisory groups Release a comprehensive written Pandemic Response and Recovery Plan
Mitigation	Focused protection of the most vulnerable: a plan for long-term care homes and for those in the community aged ≥60 years with multiple comorbidities Plans for socially vulnerable groups: e.g., temporary housing support to reduce household crowding	Voluntary focused protection: understand that the risk for those aged <60 years is similar to that from seasonal influenza
Response	Ensure critical infrastructure is ready for people who get sick, including new surge capacity in hospitals so that continuity of the medical system is ensured Ensure equitable access to healthcare	Removal of fear of SARS-CoV-2 and of each other: ensure understanding of risk in relation to other daily risks, by age group and comorbidity Removal of fear of future use of NPIs: ensure understanding of accumulated evidence about trade-offs and efficacy to end talk of future mandated lockdowns, quarantine of exposed people, school closures, community masking, and border closures Establish capabilities for endemic SARS-CoV-2: new healthcare surge capacity without plans to sacrifice healthcare for all other conditions
Recovery	Reduce fear with daily information presented with context including plans for surge capacity, give hospitalizations and death numbers with denominators, by age group, in comparison to other risks causing deaths annually, and without a focus on raw case counts Give evidence on the cost-benefit balance of NPIs and lockdowns: explain the difficult trade-offs involved and the justification for focused protection	Develop a detailed plan to overcome the impacts from the use of fear and NPIs/lockdowns on mental health, societal health, our children's education and development, missed/delayed diagnosis and treatment of medical conditions, government debt, confidence in the economy, etc Replace fear with confidence by using the EM process, with cost-benefit analysis of all recovery options open, improved communication, and a written plan that is transparently demonstrably justified by due diligence

*References: Joffe and Redman (6), Redman (363), and Redman (364)*.

## Discussion

### Possible ways forward

Governments and public health authorities worldwide have imposed their decisions, while having trouble using evidence-based policy and decision making (13, 359, 366). This has harmed many groups in society (10, 367). Many scientists also went along with the narrative that the most aggressive NPIs were necessary for the greater good, for instance, experts advising on how to modify behavior [e.g., (366, 368)]. Others have pointed out that the debate has been highly polarized and should ideally be more open-minded and nuanced (369). Society has fallen prey to groupthink (11) with the perpetuation of dysfunctional entrenched patterns in responding to the pandemic (13). It seems more important than ever to uphold and renew important values that societies fare by, to enhance the well-being of their citizens (370). Healing society should focus on people's dignity, rights, values, and humanity (370). Concurrently, it becomes imperative to use evidence-based policy and decision making (359, 371) and reflexivity (13), as used in the EM process (363).

It is key to restore the health and well-being of the wider population, and create a positive environment in which people can thrive (46). Well-being should matter to governments (230). Next to reversing the most aggressive and ineffective policies (360, 372), the way people cope with the situation is important (10, 373). Most people seem to be negatively affected in terms of health and well-being, and personality differences may also play a role (217). People that score high on proactive personality are better at spotting opportunities and acting upon them (374). They also are better able to foresee consequences and risks inherent in actions that they take and anticipate them, affecting environmental change (375). For many people access to intangible resources such as social support, and social belonging and access to tangible resources such as income, livelihood, and access to (healthy) food have been thwarted. Loss spirals accelerate once resource losses accumulate, while resource gain cycles become weaker (194). It is easy to widen the inequality gaps, but these may take years and years to close. For instance, while it was estimated before the crisis that closing the gender gap could take up to 99.5 years, after the crisis it was estimated to take 135 years (376, 377).

### Collective healing and restoring meaning

The current situation requires collective healing [(378); cf. (379)]. While programs such as Eye Movement Desensitization and Reprocessing [EMDR; (380)], brainspotting (381) and neurosculpting (382) may be effective for relieving (complex) trauma [for reviews see (383, 384)], more scalable positive psychology solutions are needed (230). Many people will feel the need to reinstate a sense of meaning in life (46). Scalable solutions may entail for instance life crafting (reflecting and setting goals and undertaking actions for important areas of life) to find meaning in life, as a written guided online intervention (385), or *via* a chatbot [e.g., (386, 387)]. Gratitude and grit may restore a sense of meaning in life and have been related to decreased suicidal ideations (388). Gratitude and well-being are correlated (389), and the connection between these seems to entail social connectedness and meaning in life (390). Communities could investigate possibilities to help many people *via* scalable solutions (10, 13, 46). For instance, life crafting and other positive psychology and mental health interventions delivered online or *via* a chatbot, could be a scalable solution and “first aid” for people experiencing issues such as anxiety, depression, and loss of purpose in life (46, 386). Goalsetting also seems promising in terms of reducing the gender and ethnic minority achievement gap for specific student populations (391). Interventions should be rigorously tested for effectiveness and they should preferably be done in concert with other positive psychology interventions tackling educational inequalities [see (392)]. Moreover, it is advisable to radically increase the voluntariness of measures. Giving people a choice instead of forcing policies upon them, might increase intervention effectiveness. For instance, when people work from home voluntarily, they experience fewer adverse effects of teleworking [e.g., (393)].

Increasing diverse citizen engagement in (global) problems (86), and grassroots movements may help counter authoritarian tendencies associated with the pandemic response, salvage democracy (151, 394–396), and increase democratization of companies post-COVID-19 (397). It may be better to strengthen people's sense of responsibility to take action after carefully laying out the pros and cons of behavior (398). Finally, we should acknowledge that for many of the proposed interventions, we would benefit from having stronger evidence from large (cluster) randomized trials, to understand whether they may work in different populations and circumstances. While the pandemic led to thousands of randomized trials of drugs, biologics, and vaccines (399, 400), few trials were performed on NPIs (401) and the research agenda on psychological and social-level interventions was even thinner. This deficiency should be remedied.

## Conclusion

As the COVID-19 crisis and NPIs of unprecedented severity and duration are related to many negative side effects and increase inequalities worldwide (402), stress, health, and trauma for vulnerable populations must be addressed (403). The economic fall-out and rise in inequalities may be long-term (403). Governments should take well-being as a spearhead for decision-making in the upcoming years (230). Hopefully, with effective interventions, the tide may be turned.

## Author contributions

MS played the primary role in the conception of the manuscript, writing, reviewing, and revising the manuscript. JI contributed to writing the manuscript, identifying studies on inequalities, and editing the manuscript. AJ wrote section on “Could we have done better”, crafted Tables 2, 3, contributed to writing, and editing the manuscript. All authors contributed to the article and approved the submitted version.

## Conflict of interest

The authors declare that the research was conducted in the absence of any commercial or financial relationships that could be construed as a potential conflict of interest.

## Publisher's note

All claims expressed in this article are solely those of the authors and do not necessarily represent those of their affiliated organizations, or those of the publisher, the editors and the reviewers. Any product that may be evaluated in this article, or claim that may be made by its manufacturer, is not guaranteed or endorsed by the publisher.

## Supplementary material

Watch here Mass formation and rising inequalities, a proposed way forward - Michaéla Schippers - YouTube the interview MS gave about this article for the podcast “Follow the Science”.

## References

[1] Adler E Hebel-Sela S Leshem OA Levy J Halperin E. A social virus: intergroup dehumanization and unwillingness to aid amidst COVID-19 – Who are the main targets? Int J Intercult Relat. (2022) 86:109–21. 10.1016/j.ijintrel.2021.11.006 34866714 PMC8629726

[2] Biesma RG Brugha R Harmer A Walsh A Spicer N Walt G. The effects of global health initiatives on country health systems: a review of the evidence from HIV/AIDS control. Health Policy Plan. (2009) 24:239–252. 10.1093/heapol/czp025 19491291 PMC2699244

[3] Jedwab R Khan AM Russ J Zaveri ED. Epidemics, pandemics, and social conflict: lessons from the past and possible scenarios for COVID-19. World Develop. (2021) 147:105629. 10.1016/j.worlddev.2021.105629 34866756 PMC8633882

[4] WHO. Working Together for Better Health and Well-Being for All: Fifth High-Level Meeting of Small Countries Reykjavik, Iceland. Regional Office for Europe: WHO (2018). Available online at: https://apps.who.int/iris/handle/10665/345576

[5] Kraaijeveld SR. COVID-19: against a lockdown approach. Asian Bioethics Rev. (2021) 13:195–212. 10.1007/s41649-020-00154-y 33262838 PMC7687977

[6] Joffe AR Redman D. The SARS-CoV-2 pandemic in high income countries such as canada: a better way forward without lockdowns. Front Public Health. (2021) 9:715904. 10.3389/fpubh.2021.715904 34926364 PMC8672418

[7] Fögen Z. The foegen effect: a mechanism by which facemasks contribute to the COVID-19 case fatality rate. Medicine. (2022) 101:e28924. 10.1097/MD.0000000000028924 35363218 PMC9282120

[8] Guerra DD Guerra DJ. Mask mandate and use efficacy for COVID-19 containment in US States. Cold Spring Harbor Lab. (2021). 10.1101/2021.05.18.21257385

[9] Panneer S Kantamaneni K Akkayasamy VS Susairaj AX Panda PK Acharya SS. The great lockdown in the wake of COVID-19 and its implications: lessons for low and middle-income countries. Int J Environ Res Public Health. (2022) 19:610. 10.3390/ijerph19010610 35010863 PMC8744631

[10] Schippers MC. For the greater good? The devastating ripple effects of the Covid-19 crisis. Front Psychol. (2020) 11:2626. 10.3389/fpsyg.2020.577740 33132987 PMC7550468

[11] Joffe AR. COVID-19: rethinking the lockdown groupthink. Front Public Health. (2021) 9:625778. 10.3389/fpubh.2021.625778 33718322 PMC7952324

[12] Melnick ER Ioannidis JPA. Should governments continue lockdown to slow the spread of covid-19? BMJ. (2020) 369:m1924. 10.1136/bmj.m1924 32493767

[13] Schippers MC Rus DC. Optimizing decision-making processes in times of COVID-19: using reflexivity to counteract information-processing failures. Front Psychol. (2021) 12:650525. 10.3389/fpsyg.2021.650525 34239479 PMC8258315

[14] Aspachs O Durante R Graziano A Mestres J Reynal-Querol M Montalvo JG. Tracking the impact of COVID-19 on economic inequality at high frequency. PLoS ONE. (2021) 16:e0249121. 10.1371/journal.pone.0249121 33788886 PMC8012053

[15] Binns C Low WY. The rich get richer and the poor get poorer: the inequality of COVID-19. Asia Pacific J Public Health. (2021) 33:185–187. 10.1177/10105395211001662 33759590

[16] Herby J Jonung L Hanke S. A literature review and meta-analysis of the effects of lockdowns on COVID-19 mortality. Stud Appl Econ. (2022) 15.

[17] Bhattacharya J Lockdowns are the 'Single Biggest Mistake in Public Health History': Stanford Medical Professor. LifeSite (2022). Retrieved from https://www.lifesitenews.com/news/lockdowns-are-the-single-biggest-mistake-in-public-health-history-stanford-medical-professor/ (accessed July 30, 2021).

[18] Hevia C, Neumeyer, PA,. A Perfect Storm: COVID-19 in Emerging Economies. VoxEU CEPR Policy Portal (2020). Available online at: https://voxeu.org/article/perfect-stormcovid-19-emerging-economies

[19] Chowdhury P Paul SK Kaisar S Moktadir MA. COVID-19 pandemic related supply chain studies: a systematic review. Transport Res Part E Logist Transport Rev. (2021) 148:102271. 10.1016/j.tre.2021.102271 33613082 PMC7881707

[20] Guan D Wang D Hallegatte S Davis SJ Huo J Li S. Global supply-chain effects of COVID-19 control measures. Nat Hum Behav. (2020) 4:577–87. 10.1038/s41562-020-0896-8 32493967

[21] Singh S Kumar R Panchal R Tiwari MK. Impact of COVID-19 on logistics systems and disruptions in food supply chain. Int J Product Res. (2021) 59:1993–2008. 10.1080/00207543.2020.1792000

[22] Santomauro DF Mantilla Herrera AM Shadid J Zheng P Ashbaugh C Pigott DM. Global prevalence and burden of depressive and anxiety disorders in 204 countries and territories in 2020 due to the COVID-19 pandemic. Lancet. (2021) 398:1700–12. 10.1016/S0140-6736(21)02143-7 34634250 PMC8500697

[23] Taquet M Holmes EA Harrison PJ. Depression and anxiety disorders during the COVID-19 pandemic: knowns and unknowns. Lancet. (2021) 398:1665–6. 10.1016/S0140-6736(21)02221-2 34634251 PMC9754944

[24] Yonzan N, Lakner, C, Gerszon Mahler, D, Aguilar, RAC, Wu, H,. Here's How Many People Covid-19 Could Push Into Poverty, According to the World Bank. World Economic Forum (2022). Retrieved from https://www.weforum.org/agenda/2020/11/covid-19-global-poverty-inequality-un-economics-coronavirus-pandemic/ (accessed May 19, 2020).

[25] Paslakis G Dimitropoulos G Katzman DK. A call to action to address COVID-19–induced global food insecurity to prevent hunger, malnutrition, eating pathology. Nutr Rev. (2020) 79:114–6. 10.1093/nutrit/nuaa069 32651592 PMC7454780

[26] Zetzsche DA. One million or one hundred million casualties? The impact of the COVID-19 crisis on low-and middle-income countries. SSRN. (2020) 2020–008. 10.2139/ssrn.3597657

[27] Oxfam. The Hunger Virus Multiplies: Deadly Recipe of Conflict, COVID-19 and Climate Accelerate World Hunger. Oxfam (2021). Available online at: https://www.oxfam.org/en/research/hunger-virus-multiplies-deadly-recipe-conflictcovid-19-and-climate-accelerate-world (accessed August 8, 2022).

[28] Nelson EM Nisbett N Gillespie S. Historicising global nutrition: critical reflections on contested pasts and reimagined futures. BMJ Glob Health. (2021) 6:e006337. 10.1136/bmjgh-2021-006337 34772690 PMC8593273

[29] Jones AD. Food insecurity and mental health status: a global analysis of 149 countries. Am J Prev Med. (2017) 53:264–73. 10.1016/j.amepre.2017.04.008 28457747

[30] Nanath K Balasubramanian S Shukla V Islam N Kaitheri S. Developing a mental health index using a machine learning approach: assessing the impact of mobility and lockdown during the COVID-19 pandemic. Technol Forecast Soc Change. (2022) 178:121560. 10.1016/j.techfore.2022.121560 35185222 PMC8841156

[31] Storm S. Lessons for the age of consequences: COVID-19 and the macroeconomy. Rev Polit Econ. (2021) 1–40. 10.1080/09538259.2021.1985779

[32] Wachtler B Michalski N Nowossadeck E Diercke M Wahrendorf M Santos-Hövener C. Socioeconomic inequalities and COVID-19 – a review of the current international literature. J Health Monit. (2020) 3 (Suppl. 7):3–17. 10.25646/7059 35146298 PMC8734114

[33] Civilsdaily. What Rising Inequality Means. Civilsdaily (2021). Retrieved from https://www.civilsdaily.com/news/what-rising-inequality-means/ (accessed May 19, 2022).

[34] Deshmukh A,. This Simple Chart Reveals the Distribution of Global Wealth. Visual Capitalist (2022). Retrieved from https://www.visualcapitalist.com/distribution-of-global-wealth-chart/ (accessed May 19, 2021).

[35] Calhoun JB Death Squared: The Explosive Growth Demise of a Mouse Population, Vol 60. SAGE Publications. (1973). p. 80–8. Available online at: https://web.archive.org/web/20191122022742id_/https://journals.sagepub.com/doi/pdf/10.1177/00359157730661P202 10.1177/00359157730661P202 PMC1644264 4734760

[36] Sapolsky RM. The influence of social hierarchy on primate health. Science. (2005) 308:648–52. 10.1126/science.1106477 15860617

[37] Sapolsky RM Share LJ. Emergence of a peaceful culture in wild baboons. PLOS Biol. (2004) 2:e124. 10.1371/journal.pbio.0020106 15094808 PMC387274

[38] Smith GD Shipley MJ Rose G. Magnitude and causes of socioeconomic differentials in mortality: further evidence from the whitehall study. J Epidemiol Commun Health. (1990) 44:265–70. 2277246 10.1136/jech.44.4.265 PMC1060667

[39] Snyder-Mackler N Burger JR Gaydosh L Belsky DW Noppert GA Campos FA. Social determinants of health and survival in humans and other animals. Science. (2020) 368:eaax9553. 10.1126/science.aax9553 32439765 PMC7398600

[40] Chen E Miller GE. Socioeconomic status and health: mediating and moderating factors. Annu Rev Clin Psychol. (2013) 9:723–49. 10.1146/annurev-clinpsy-050212-185634 23245339

[41] Marmot M Wilkinson R. Social Determinants of Health. Oxford: Oxford University Press (2005). 10.1093/acprof:oso/9780198565895.001.0001

[42] Bajos N Jusot F Pailhé A Spire A Martin C Meyer L. When lockdown policies amplify social inequalities in COVID-19 infections: evidence from a cross-sectional population-based survey in France. BMC Public Health. (2021) 21:705. 10.1186/s12889-021-10521-5 33845798 PMC8040364

[43] Plott CF Kachalia AB Sharfstein JM. Unexpected health insurance profits and the COVID-19 crisis. JAMA. (2020) 324:1713–4. 10.1001/jama.2020.19925 33141199

[44] Baines J Hager SB. The great debt divergence and its implications for the Covid-19 crisis: mapping corporate leverage as power. New Polit Econ. (2021) 26:885–901. 10.1080/13563467.2020.1865900

[45] Kira IA Shuwiekh HAM Alhuwailah A Ashby JS Sous Fahmy Sous M Baali SBA. The effects of COVID-19 and collective identity trauma (intersectional discrimination) on social status and well-being. Traumatology. (2021) 27:29–39. 10.1037/trm0000289

[46] de Jong EM Ziegler N Schippers MC. From shattered goals to meaning in life: life crafting in times of the COVID-19 pandemic. Front Psychol. (2020) 11:577708. 10.3389/fpsyg.2020.577708 33178081 PMC7593511

[47] Desmet M. The Psychology of Totalitarianism. White River Junction, VT: Chealsea Green Publishing (2022).

[48] Cheng Z Mendolia S Paloyo AR Savage DA Tani M. Working parents, financial insecurity, and childcare: mental health in the time of COVID-19 in the UK. Rev Econ Househ. (2021) 19:123–44. 10.1007/s11150-020-09538-3 33456425 PMC7802611

[49] Krauss JE Artur L Brockington D Castro E Fernando J Fisher J. ‘To prevent this disease, we have to stay at home, but if we stay at home, we die of hunger' – livelihoods, vulnerability and coping with Covid-19 in rural Mozambique. World Dev. (2022) 151, 105757. 10.1016/j.worlddev.2021.105757 34848914 PMC8612814

[50] Corman H Noonan K Reichman NE Schultz J. Effects of financial insecurity on social interactions. J Socio Econ. (2012) 41:574–83. 10.1016/j.socec.2012.05.006

[51] Dickerson J Kelly B Lockyer B Bridges S Cartwright C Willan K. ‘When will this end? Will it end?' The impact of the March–June 2020 UK COVID-19 lockdown response on mental health: a longitudinal survey of mothers in the Born in Bradford study. BMJ Open. (2022) 12:e047748. 10.1136/bmjopen-2020-047748 35017230 PMC8753090

[52] Polsky JY Gilmour H. Food insecurity and mental health during the COVID-19 pandemic. Health reports. (2020) 31:3–11. 10.25318/82-003-x202001200001 33325672

[53] Vermote B Waterschoot J Morbée S Van der Kaap-Deeder J Schrooyen C Soenens B. do psychological needs play a role in times of uncertainty? Associations with well-being during the COVID-19 crisis. J Happin Stud. (2022) 23:257–83. 10.1007/s10902-021-00398-x 33942013 PMC8081282

[54] Aledort JE Lurie N Wasserman J Bozzette SA. Non-pharmaceutical public health interventions for pandemic influenza: an evaluation of the evidence base. BMC Public Health. (2007) 7:208. 10.1186/1471-2458-7-208 17697389 PMC2040158

[55] WHO. Non-Pharmaceutical Public Health Measures for Mitigating the Risk and Impact of Epidemic and Pandemic Influenza: Annex: Report of Systematic Literature Reviews. WHO (2019). Available at: https://apps.who.int/iris/bitstream/handle/10665/329439/WHO-WHE-IHM-GIP-2019.1-eng.pdf

[56] WHO. Strengthening and Adjusting Public Health Measures Throughout the COVID-19 Transition Phases: Policy Considerations for the WHO European Region, 24 April 2020. Copenhagen: WHO (2020).

[57] Ioannidis JPA. Coronavirus disease 2019: the harms of exaggerated information and non-evidence-based measures. Eur J Clin Invest. (2020) 50:e13222. 10.1111/eci.13222 32191341 PMC7163529

[58] Edmonds B. Review of Critical Mass: How One Thing Leads to Another. New York, NY: Farrar, Straus and Giroux (2006).

[59] Le Bon G. The Crowd: A Study of the Popular Mind. New York, NY: Courier Corporation (2002).

[60] Kok VJ Lim MK Chan CS. Crowd behavior analysis: a review where physics meets biology. Neurocomputing. (2016) 177:342–62. 10.1016/j.neucom.2015.11.021

[61] Bor A Jørgensen FJ Petersen MB. Prejudice Against the Vaccinated and the Unvaccinated During the COVID-19 Pandemic: A Global Conjoint Experiment. (2022). 10.31234/osf.io/t2g45

[62] Bylund PL Packard MD. Separation of power and expertise: Evidence of the tyranny of experts in Sweden's COVID-19 responses. South Econ J. (2021) 87:1300–19. 10.1002/soej.12493 33821054 PMC8014802

[63] Drury J Carter H Ntontis E Guven ST. Public behaviour in response to the COVID-19 pandemic: understanding the role of group processes. BJPsych Open. (2021) 7:e11. 10.1192/bjo.2020.139 33283693 PMC7844149

[64] Prentice C Quach S Thaichon P. Antecedents and consequences of panic buying: the case of COVID-19. Int J Consum Stud. (2022) 46:132–46. 10.1111/ijcs.12649

[65] Bavel JJV Baicker K Boggio PS Capraro V Cichocka A Cikara M. Using social and behavioural science to support COVID-19 pandemic response. Nat Hum Behav. (2020) 4:460–71. 10.1038/s41562-020-0884-z 32355299

[66] Rayamajhee V Paniagua P. Coproduction and the crafting of cognitive institutions during the COVID-19 pandemic. J Inst Econ. (2022) 1–7. 10.1017/S1744137422000078

[67] Krings VC Steeden B Abrams D Hogg MA. Social attitudes and behavior in the COVID-19 pandemic: evidence and prospects from research on group processes and intergroup relations. Group Process Intergroup Relat. (2021) 24:195–200. 10.1177/1368430220986673

[68] Le Bon G editor. Psychology of the Great War: The First World War and Its Origins. New York, NY: Routledge (2018).

[69] Postmes T Spears R. Deindividuation and antinormative behavior: a meta-analysis. Psychol Bull. (1998) 123:238.

[70] Abrams D Lalot F Hogg MA. Intergroup and intragroup dimensions of COVID-19: a social identity perspective on social fragmentation and unity. Group Process Intergroup Relat. (2021) 24:201–9. 10.1177/1368430220983440

[71] Abrams D Travaglino GA Marques JM Davies B Randsley de Moura G. Collective deviance: scaling up subjective group dynamics to superordinate categories reveals a deviant ingroup protection effect. J Pers Soc Psychol. (2021) 123:353–72. 10.1037/pspi0000356 33475399

[72] Wiggins JA Dill F Schwartz RD. On “status-liability”. Sociometry. (1965) 28:197–209. 14331852

[73] Grant PR Smith HJ. Activism in the time of COVID-19. Group Process Intergr Relat. (2021) 24:297–305. 10.1177/1368430220985208

[74] Sharp G. From Dictatorship to Democracy. Boston, MA: The Albert Einstein Institution (2010).

[75] Chirisa I Mutambisi T Chivenge M Mabaso E Matamanda AR Ncube R. The urban penalty of COVID-19 lockdowns across the globe: manifestations and lessons for anglophone sub-Saharan Africa. GeoJournal. (2022) 87:815–28. 10.1007/s10708-020-10281-6 32868960 PMC7450483

[76] Spring C Garthwaite K Fisher A. Containing hunger, contesting injustice? Exploring the transnational growth of foodbanking- and counter-responses- before and during the COVID-19 pandemic. Food Ethics. (2022) 7:6. 10.1007/s41055-022-00099-y 35340530 PMC8934159

[77] Mayseless O Popper M. Reliance on leaders and social institutions: an attachment perspective. Attach Hum Dev. (2007) 9:73–93. 10.1080/14616730601151466 17364483

[78] Volkan V. Blind Trust: Large Groups and Their Leaders in Times of Crisis and Terror. Richmond, VA: Pitchstone Publishing (2014).

[79] Antonakis J. Leadership to defeat COVID-19. Group Process Intergroup Relat. (2021) 24:210–5. 10.1177/1368430220981418

[80] Binagwaho A. We need compassionate leadership management based on evidence to defeat COVID-19. Int J Health Policy Manage. (2020) 9:413–4. 10.34172/ijhpm.2020.73 32610733 PMC7719216

[81] Gupta S Simon KI Wing C. Mandated and Voluntary Social Distancing During the Covid-19 Epidemic: A Review. New York, NY: National Bureau of Economic Research (2020). 10.3386/w28139

[82] Schmelz K Bowles S. Opposition to voluntary and mandated COVID-19 vaccination as a dynamic process: evidence and policy implications of changing beliefs. Proc Natl Acad Sci USA. (2022) 119:e2118721119. 10.1073/pnas.2118721119 35316133 PMC9060490

[83] Yan Y Malik AA Bayham J Fenichel EP Couzens C Omer SB. Measuring voluntary and policy-induced social distancing behavior during the COVID-19 pandemic. Proc Natl Acad Sci USA. (2021) 118:e2008814118. 10.1073/pnas.2008814118 33820846 PMC8076999

[84] Teichman D Underhill K. Infected by bias: behavioral science and the legal response to COVID-19. Am J Law Med. (2021) 47:205–48. 10.1017/amj.2021.16 34405780

[85] Schmelz K. Enforcement may crowd out voluntary support for COVID-19 policies, especially where trust in government is weak and in a liberal society. Proc Natl Acad Sci USA. (2021) 118:e2016385118. 10.1073/pnas.2016385118 33443149 PMC7817206

[86] Carpini MXD Cook FL Jacobs LR. Public deliberation, discursive participation, and citizen engagement: a review of the empirical literature. Ann Rev Polit Sci. (2004) 7:315–. 10.1146/annurev.polisci.7.121003.091630

[87] Allen DW. Covid-19 lockdown cost/benefits: a critical assessment of the literature. Int J Econ Bus. (2022) 29:1–32. 10.1080/13571516.2021.1976051

[88] Flaxman S Mishra S Gandy A Unwin HJT Mellan TA Coupland H. Estimating the effects of non-pharmaceutical interventions on COVID-19 in Europe. Nature. (2020). 584:257–61. 10.1038/s41586-020-2405-7 32512579

[89] Siqueira CADS Freitas YNLD Cancela MDC Carvalho M Oliveras-Fabregas A de Souza DLB. The effect of lockdown on the outcomes of COVID-19 in Spain: an ecological study. PLoS ONE. (2020) 15:e0236779. 10.1371/journal.pone.0236779 32726363 PMC7390404

[90] Umer H Khan MS. Evaluating the effectiveness of regional lockdown policies in the containment of Covid-19: evidence from Pakistan. arXiv preprint. (2020) arXiv:2006.02987. 10.31219/osf.io/s3fkp

[91] Gibson J. Government mandated lockdowns do not reduce Covid-19 deaths: implications for evaluating the stringent New Zealand response. N Zeal Econ Pap. (2022) 56:17–28. 10.1080/00779954.2020.1844786

[92] Graso M Henwood A Aquino K Dolan P Chen FX. The dark side of belief in COVID-19 scientists and scientific evidence. Pers Individ Dif. (2022) 193:111594. 10.1016/j.paid.2022.111594 35291670 PMC8913370

[93] Silverman M Sibbald R Stranges S. Ethics of COVID-19-related school closures. Can J Public Health. (2020) 111:462–5. 10.17269/s41997-020-00396-1 32767271 PMC7412780

[94] McPhail C. The Myth of the Madding Crowd. New York, NY: Routledge (2017). 10.4324/9781315133270

[95] Rempel MW Fisher RJ. Perceived threat, cohesion, and group problem solving in intergroup conflict. Int J Confl Manage. (1997) 8:216–34.

[96] Fischer R Karl JA. Predicting behavioral intentions to prevent or mitigate COVID-19: a cross-cultural meta-analysis of attitudes, norms, and perceived behavioral control effects. Soc Psychol Personal Sci. (2022) 13:264–76. 10.1177/19485506211019844

[97] Graso M. The new normal: Covid-19 risk perceptions and support for continuing restrictions past vaccinations. PLoS ONE. (2022) 17:e0266602. 10.1371/journal.pone.0266602 35395026 PMC8993013

[98] Mulligan CB Arnott RD. Non-Covid Excess Deaths, 2020-21: Collateral Damage of Policy Choices? (No. w30104). National Bureau of Economic Research (2022). 10.3386/w30104

[99] Arendt H. The Origins of Totalitarianism [1951]. New York, NY (1973).

[100] Riek BM Mania EW Gaertner SL. Intergroup threat and outgroup attitudes: a meta-analytic review. Pers Soc Psychol Rev. (2006) 10:336–53. 10.1207/s15327957pspr1004_4 17201592

[101] Haslam N Stratemeyer M. Recent research on dehumanization. Curr Opin Psychol. (2016) 11:25–9. 10.1016/j.copsyc.2016.03.009

[102] Graso M Chen FX Reynolds T. Moralization of Covid-19 health response: asymmetry in tolerance for human costs. J Exp Soc Psychol. (2021) 93:104084. 10.1016/j.jesp.2020.104084 33311735 PMC7717882

[103] Becker JC Wagner U Christ O. Consequences of the 2008 financial crisis for intergroup relations:The role of perceived threat and causal attributions. Group Proc Intergroup Relat. (2011) 14:871–85. 10.1177/1368430211407643

[104] Fritsche I Jonas E Kessler T. Collective reactions to threat: implications for intergroup conflict and for solving societal crises. Soc Issues Policy Rev. (2011) 5:101–36. 10.1111/j.1751-2409.2011.01027.x

[105] Krosch AR Tyler TR Amodio DM. Race and recession: effects of economic scarcity on racial discrimination. J Pers Soc Psychol. (2017) 113:892–909. 10.1037/pspi0000112 28910122

[106] Shannon P. Part Two: COVID, Politics and Psychology. Quadrant (2022).

[107] Sullivan D Landau MJ Rothschild ZK. An existential function of enemyship: evidence that people attribute influence to personal and political enemies to compensate for threats to control. J Pers Soc Psychol. (2010) 98:434–49. 10.1037/a0017457 20175623

[108] Petersson BO. Hot conflict and everyday banality: enemy images, scapegoats and stereotypes. Development. (2009) 52:460–5. 10.1057/dev.2009.59

[109] Slavich GM. Social safety theory: understanding social stress, disease risk, resilience, and behavior during the COVID-19 pandemic and beyond. Curr Opin Psychol. (2022) 45:101299. 10.1016/j.copsyc.2022.101299 35219156 PMC8769662

[110] Zion SR Louis K Horii R Leibowitz K Heathcote LC Crum AJ. Making sense of a pandemic: mindsets influence emotions, behaviors, health, and wellbeing during the COVID-19 pandemic. Soc Sci Med. (2022) 301:114889. 10.1016/j.socscimed.2022.114889 35430098 PMC8930786

[111] Pyszczynski T Lockett M Greenberg J Solomon S. Terror management theory and the COVID-19 pandemic. J Hum Psychol. (2021) 61:173–89. 10.1177/0022167820959488 38603072 PMC7498956

[112] Anderson CA Bushman BJ. Human aggression. Annu Rev Psychol. (2002) 53:27–51. 10.1146/annurev.psych.53.100901.135231 11752478

[113] Killgore WDS Cloonan SA Taylor EC Anlap I Dailey NS. Increasing aggression during the COVID-19 lockdowns. J Affect Disord Rep. (2021) 5:100163. 10.1016/j.jadr.2021.100163 34075370 PMC8161773

[114] Mazza M Marano G Lai C Janiri L Sani G. Danger in danger: interpersonal violence during COVID-19 quarantine. Psychiatry Res. (2020) 289:113046. 10.1016/j.psychres.2020.113046 32387794 PMC7190494

[115] Zillmann D. Transfer of excitation in emotional behavior. In: Cacioppo JT, Petty ER, editor. SOCIAL Psychophysiology: A Sourcebook. New York, NY: Guilford (1983). p. 215–40.

[116] Bezo B Maggi S. Living in “survival mode:” intergenerational transmission of trauma from the holodomor genocide of 1932–1933 in Ukraine. Soc Sci Med. (2015) 134:87–94. 10.1016/j.socscimed.2015.04.009 25931287

[117] Brom D. Thoughts about survival mode theory of posttraumatic reactions. In: Helping Children Cope With Trauma: Individual, Family and Community Perspectives. Jerusalem: Routledge (2014). p. 243–8.

[118] Marcus-Newhall A Pedersen WC Carlson M Miller N. Displaced aggression is alive and well: a meta-analytic review. J Person Soc Psychol. (2000) 78:670. 10.1037/0022-3514.78.4.670 10794373

[119] Ye B Zeng Y Im H Liu M Wang X Yang Q. The relationship between fear of COVID-19 and online aggressive behavior: a moderated mediation model. Front Psychol. (2021) 12:589615. 10.3389/fpsyg.2021.589615 33658957 PMC7917050

[120] McGregor HA Lieberman JD Greenberg J Solomon S Arndt J Simon L. Terror management and aggression: evidence that mortality salience motivates aggression against worldview-threatening others. J Person Soc Psychol. (1998) 74:590. 9523407 10.1037//0022-3514.74.3.590

[121] Stein DH Schroeder J Hobson NM Gino F Norton MI. When alterations are violations: moral outrage and punishment in response to (even minor) alterations to rituals. J Person Soc Psychol. (2021) 123.1:123. 10.31234/osf.io/yd7tg 33492153

[122] Henderson RK Schnall S. Disease and disapproval: COVID-19 concern is related to greater moral condemnation. Evol Psychol. (2021) 19:14747049211021524. 10.1177/14747049211021524 34112018 PMC10358411

[123] Dennis D Radnitz C Wheaton MG. A perfect storm? Health anxiety, contamination fears, and COVID-19: lessons learned from past pandemics and current challenges. Int J Cogn Ther. (2021) 14:497–513. 10.1007/s41811-021-00109-7 33907592 PMC8061445

[124] Bendau A Kunas SL Wyka S Petzold MB Plag J Asselmann E. Longitudinal changes of anxiety and depressive symptoms during the COVID-19 pandemic in Germany: the role of pre-existing anxiety, depressive, and other mental disorders. J Anxiety Disord. (2021) 79:102377. 10.1016/j.janxdis.2021.102377 33662702 PMC9758512

[125] Lathabhavan R Vispute S. Examining the mediating effects of stress on fear of COVID-19 and well-being using structural equation modeling. Int J Ment Health Addict. (2021) 18:1–9. 10.1007/s11469-021-00541-y 34025306 PMC8130788

[126] Wright LJ Williams SE Veldhuijzen van Zanten JJCS. Physical Activity protects against the negative impact of coronavirus fear on adolescent mental health and well-being during the COVID-19 pandemic. Front Psychol. (2021) 12:580511. 10.3389/fpsyg.2021.580511 33776827 PMC7990778

[127] Alamilla SG Cano MÁ. COVID-19 and adverse social determinants of health. Behav Med. (2022) 48:67–71. 10.1080/08964289.2022.2027859 35318902

[128] Bambra C Lynch J Smith KE. The Unequal Pandemic: COVID-19 and Health Inequalities. Bristol: Policy Press (2021) 10.46692/9781447361251

[129] Broadbent A Streicher P. Can you lock down in a slum? And who would benefit if you tried? Difficult questions about epidemiology's commitment to global health inequalities during Covid-19. Glob Epidemiol. (2022) 4:100074. 10.1016/j.gloepi.2022.100074 35647518 PMC9125993

[130] Idler E Bernau JA Zaras D. Narratives and counter-narratives in religious responses to COVID-19: a computational text analysis. PLOS ONE. (2022) 17:e0262905. 10.1371/journal.pone.0262905 35113914 PMC8812967

[131] Khoury MJ Ioannidis JPA. Big data meets public health. Science. (2014) 346:1054–5. 10.1126/science.aaa2709 25430753 PMC4684636

[132] Kameda T Toyokawa W Tindale RS. Information aggregation and collective intelligence beyond the wisdom of crowds. Nat Rev Psychol. (2022) 12:8047. 10.1038/s44159-022-00054-y

[133] Hughes AC Orr MC Ma K Costello MJ Waller J Provoost P. Sampling biases shape our view of the natural world. Ecography. (2021) 44:1259–69. 10.1111/ecog.05926

[134] Pleyers G. The pandemic is a battlefield. Social movements in the COVID-19 lockdown. J Civil Soc. (2020) 16:295–312. 10.1080/17448689.2020.1794398

[135] Greer J Fitzgerald K Vijaykumar S. Narrative Elaboration Makes Regarding COVID-19 More Believable. Misinformation and Corrective Information. (2022). 10.21203/rs.3.rs-1461954/v1 35765114 PMC9241297

[136] Godlee F. Why healthcare needs rebels. BMJ. (2021) 375:n2559. 10.1136/bmj.n2559

[137] Kaufmann E. Academic freedom in crisis: punishment, political discrimination, and self-censorship. Cent Study Partisansh Ideol. (2021) 2:1–195. Available online at: https://www.hoplofobia.info/wp-content/uploads/2021/05/2021-Academic-Freedom-in-Crisis.pdf

[138] Sunstein CR. Why Societies Need Dissent. Vol. 9. Cambridge, MA: Harvard University Press (2005). 10.4159/9780674267657

[139] Kavanagh MM Singh R. Democracy, capacity, and coercion in pandemic response: COVID-19 in comparative political perspective. J Health Polit Policy Law. (2020) 45:997–1012. 10.1215/03616878-8641530 32464665

[140] Gostin LO Hodge JG. US emergency legal responses to novel coronavirus: balancing public health and civil liberties. JAMA. (2020) 323:1131–2. 10.1001/jama.2020.2025 32207808

[141] World Health Organization Writing Group. Nonpharmaceutical interventions for pandemic influenza, national and community measures. Emerg Infect Dis. (2006) 12:88–94. 10.3201/eid1201.051371 16494723 PMC3291415

[142] Sly L,. Stirrings of Unrest Around the World Could Portend Turmoil as Economies Collapse. The Washington Post (2020). Available online at: https://www.washingtonpost.com/world/coronavirus-protests-lebanonindia-iraq/2020/04/19/1581dde4-7e5f-11ea-84c2-0792d8591911_story.Html (accessed June 2, 2020).

[143] Byrne S Hart PS. The boomerang effect a synthesis of findings and a preliminary theoretical framework. Ann Int Commun Assoc. (2009) 33:3–37. 10.1080/23808985.2009.11679083

[144] Cohen AR. A dissonance analysis of the boomerang effect. J Pers. (1962) 30:75–88. 13880221 10.1111/j.1467-6494.1962.tb02306.x

[145] Kean S. The Soviet Era's deadliest scientist is regaining popularity in Russia. Atlantic. (2017) 19. Available online at: https://www.theatlantic.com/science/archive/2017/12/trofim-lysenko-soviet-union-russia/548786/

[146] Kolchinsky EI Kutschera U Hossfeld U Levit GS. Russia's new lysenkoism. Curr Biol. (2017) 27:R1042–7. 10.1016/j.cub.2017.07.045 29017033

[147] Rittberger B Richardson J. What happens when we do not defend academic freedom. J Euro Public Policy. (2019) 26:324–4. 10.1080/13501763.2017.1316946

[148] Motta M. The dynamics and political implications of anti-intellectualism in the United States. Am Polit Res. (2018) 46:465–498. 10.1177/1532673X17719507

[149] Teixeira da Silva JA. How to shape academic freedom in the digital age? Are the retractions of opinionated papers a prelude to “cancel culture” in academia? Curr Res Behav Sci. (2021) 2:100035. 10.1016/j.crbeha.2021.100035

[150] Della Porta D. How progressive social movements can save democracy in pandemic times. Interface. (2020) 12:355–8. Retrived from: https://www.interfacejournal.net/wp-content/uploads/2020/07/Interface-12-1-Della-Porta.pdf

[151] Ioannidis J, Schippers, M,. Saving Democracy From the Pandemic. Tablet Magazine (2022). Retrieved from https://www.tabletmag.com/sections/science/articles/saving-democracy-from-pandemic (accessed May 19, 2022).

[152] Daly TG. The pandemic and the future of global democracy. In: Routledge Handbook of Law and the COVID-19 Pandemic. New York, NY: Routledge (2022). p. 5–17. 10.4324/9781003211952-2

[153] Della Porta D. How Social Movements Can Save Democracy: Democratic Innovations From Below. Cambridge: John Wiley and Sons (2020).

[154] Seedhouse D. The Case for Democracy in the COVID-19 Pandemic. Sage (2020). 10.4135/9781529757163

[155] Ioannidis JP. Why most published research findings are false. PLoS Med. (2005) 2:e124. 10.1371/journal.pmed.0020124 16060722 PMC1182327

[156] Carty V. New information communication technologies and grassroots mobilization. Inform Commun Soc. (2010) 13:155–73. 10.1080/1369118090291565833788791

[157] Carty V Onyett J. Protest, cyberactivism and new social movements: the reemergence of the peace movement post 9/11. Soc Mov Stud. (2006) 5:229–49. 10.1080/14742830600991586

[158] Fournier V. Utopianism and the cultivation of possibilities: grassroots movements of hope. Sociol Rev. (2002) 50 (1_suppl):189–216. 10.1111/j.1467-954X.2002.tb03585.x

[159] Goodwin J Jasper J Polletta F. The return of the repressed: the fall and rise of emotions in social movement theory. Mobilization. (2006) 5:65–83. 10.17813/maiq.5.1.74u39102m107g748

[160] Gulliver R Wibisono S Fielding KS Louis WR. The Psychology of Effective Activism. Cambridge: Cambridge University Press (2021). 10.1017/9781108975476

[161] Roy N. Conclusion: will civil resistance work? In: Nonviolent Resistances in the Contemporary World. Routledge (2021). p. 133–6. 10.4324/9781003109310-6

[162] Moyer B MacAllister J Soifer MLFS. Doing Democracy: The MAP Model for Organizing Social Movements. Gabriola, BC: New Society Publishers (2001).

[163] Hamilton LC Safford TG. Elite cues and the rapid decline in trust in science agencies on COVID-19. Sociol Perspect. (2021) 64:988–1011. 10.1177/07311214211022391

[164] Sovacool BK Dunlap A. Anarchy, war, or revolt? Radical perspectives for climate protection, insurgency and civil disobedience in a low-carbon era. Energy Res Soc Sci. (2022) 86:102416. 10.1016/j.erss.2021.102416

[165] Müller-Bachmann E Chorvát I Mefalopulos A. Heading for a better world: micropolitical activism of young people seeking social change. J Youth Stud. (2022) 1–19. 10.1080/13676261.2022.2053669

[166] Fraser N. Why overcoming prejudice is not enough: a rejoinder to richard rorty. Crit Horiz. (2000) 1:21–8. 10.1163/156851600510408

[167] Maguire ER. Policing, state repression, and the pro-democracy movement in Hong Kong. Policing. (2020) 14:840–2. 10.1093/police/paaa077

[168] Mayer NZ Bert U. Movement and countermovement interaction: mobilization, tactics, state involvement. In: Social Movements in an Organizational Society. Oxfordshire: Routledge (2017). 10.4324/9781315129648-10

[169] Bennoune K. “Lest we should sleep”: COVID-19 and human rights. Am J Int Law. (2020) 114:666–76. 10.1017/ajil.2020.68

[170] Stott C Radburn M. Understanding crowd conflict: social context, psychology and policing. Curr Opin Psychol. (2020) 35:76–80. 10.1016/j.copsyc.2020.03.001 32371380

[171] Gelderloos P,. How Nonviolence Protects the State. Cambridge: South End Press (2007). Available at: https://mirror.anarhija.net/lib.anarhija.net/mirror/p/pg/peter-gelderloos-how-nonviolence-protects-the-state.c117.pdf

[172] Janecka IP. Democracy is failing, health of nations is failing, and pandemic is raging: systems science exposés. Am J Educ Res. (2021) 9:300–12. 10.12691/education-9-5-8

[173] Alperstein N. Conflict and contentiousness: network connections and pockets of resistance in social movements. In: Performing Media Activism in the Digital Age. Springer (2021). 10.1007/978-3-030-73804-4_4

[174] Sternisko A Cichocka A Van Bavel JJ. The dark side of social movements: social identity, non-conformity, and the lure of conspiracy theories. Curr Opin Psychol. (2020) 35:1–6. 10.1016/j.copsyc.2020.02.007 32163899

[175] Darius P Urquhart M. Disinformed social movements: a large-scale mapping of conspiracy narratives as online harms during the COVID-19 pandemic. Online Soc Netw Media. (2021) 26:100174. 10.1016/j.osnem.2021.100174 34642647 PMC8495371

[176] Loadenthal M. The politics of attack: communiqués and insurrectionary violence. I: The Politics of Attack. Manchester: Manchester University Press (2017). 10.7228/manchester/9781526114457.001.0001

[177] Stephan MJ Chenoweth E. Why civil resistance works: the strategic logic of nonviolent conflict. Int Secur. (2008) 33:7–44. 10.1162/isec.2008.33.1.7

[178] Chenoweth E Stephan MJ Stephan M. Why Civil Resistance Works: The Strategic Logic of Nonviolent Conflict. New York, NY: Columbia University Press.

[179] Kraemer KR. Strategic nonviolent struggle in the twenty first century. J Soc Encount. (2021) 5:51–4. Available at: https://digitalcommons.csbsju.edu/social_encounters/vol5/iss1/9

[180] Pagnucco R. Review of civil resistance: what everyone needs to know. J Soc Encount. (2022) 6:177–81. Available online at: Available at: https://digitalcommons.csbsju.edu/social_encounters/vol6/iss1/21

[181] Hallward M Masullo J Mouly C. Civil resistance in armed conflict: leveraging nonviolent action to navigate war, oppose violence and confront oppression. J Peacebuild Dev. (2017) 12:1–9. 10.1080/15423166.2017.1376431

[182] Chenoweth E. Civil Resistance: What Everyone Needs to Know^®^. New York, NY: Oxford University Press (2021).

[183] Said E. The Public Role of Writers and Intellectuals. Vol. 10. Princeton, NJ; Oxford Princeton University Press (2005). 10.1515/9781400826681.15

[184] Shahinpoor N Matt BF. The power of one: dissent and organizational life. J Bus Ethics. (2007) 74:37–48. 10.1007/s10551-006-9218-y

[185] Della Porta D. Protests as critical junctures: some reflections towards a momentous approach to social movements. Soc Mov Stud. (2020) 19:556–75. 10.1080/14742837.2018.1555458

[186] Ando M Furuichi M. The association of COVID-19 employment shocks with suicide and safety net use: an early-stage investigation. PLoS ONE. (2022) 17:e0264829. 10.1371/journal.pone.0264829 35324902 PMC8947077

[187] Hirschberger G. Collective trauma and the social construction of meaning. Front Psychol. (2018) 9:1441. 10.3389/fpsyg.2018.01441 30147669 PMC6095989

[188] Erikson K. Everything in Its Path. New York, NY: Simon and Schuster (1976).

[189] Maitlis S Sonenshein S. Sensemaking in crisis and change: inspiration and insights from Weick (1988). J Manage Stud. (2010) 47:551–80. 10.1111/j.1467-6486.2010.00908.x

[190] Stanley ML Barr N Peters K Seli P. Analytic-thinking predicts hoax beliefs and helping behaviors in response to the COVID-19 pandemic. Think Reason. (2021) 27:464–77. 10.1080/13546783.2020.1813806

[191] Basinger ED Wehrman EC McAninch KG. Grief communication and privacy rules: examining the communication of individuals bereaved by the death of a family member. J Fam Commun. (2016) 16:285–302. 10.1080/15267431.2016.1182534

[192] Hobfoll SE. Conservation of resources: a new attempt at conceptualizing stress. Am Psychol. (1989) 44:513–24. 2648906 10.1037//0003-066x.44.3.513

[193] Hobfoll SE. Conservation of resources theory: its implication for stress, health, and resilience. In: The Oxford Handbook of Stress, Health, and Coping. Oxford University Press (2011). 10.1093/oxfordhb/9780195375343.013.0007

[194] Hobfoll SE Halbesleben J Neveu JP Westman M. Conservation of resources in the organizational context: the reality of resources and their consequences. Ann Rev Organ Psychol Organ Behav. (2018) 5:103–28. 10.1146/annurev-orgpsych-032117-104640

[195] Fuller B Marler LE. Change driven by nature: a meta-analytic review of the proactive personality literature. J Vocat Behav. (2009) 75:329–45. 10.1016/j.jvb.2009.05.008

[196] Hobfoll SE. The influence of culture, community, and the nested-self in the stress process: advancing conservation of resources theory. Appl Psychol. (2001) 50:337–421. 10.1111/1464-0597.00062

[197] Peck JA. The disproportionate impact of COVID-19 on women relative to men: a conservation of resources perspective. Gend Work Organ. (2021) 28:484–97. 10.1111/gwao.12597

[198] Naranjo AM, Sun, Q,. Women affected most by covid-19 disruptions in the Labor Market: St. Louis Fed. Saint Louis Fed Eagle (2022). Available online at https://www.stlouisfed.org/publications/regional-economist/first-quarter-2021/women-affected-most-covid-19-disruptions-labor-market

[199] Rosenfeld DL Tomiyama AJ. Can a pandemic make people more socially conservative? Political ideology, gender roles, and the case of COVID-19. J Appl Soc Psychol. (2021) 51:425–33. 10.1111/jasp.12745 33821034 PMC8014651

[200] Shelef L Schiff M Pat-Horenczyk R Dekel R. COVID-19 vs. terrorism: contribution of the COR theory to the process of coping with invisible threats. J Psychiatr Res. (2022) 147:176–82. 10.1016/j.jpsychires.2022.01.023 35051716 PMC8753990

[201] Kalinowski S Łuczak A Koziolek A. The social dimension of security: the dichotomy of respondentsandrsquo; perceptions during the COVID-19 pandemic. Sustainability. (2022) 14:1363. 10.3390/su14031363

[202] Crayne MP. The traumatic impact of job loss and job search in the aftermath of COVID-19. Psychol Trauma Theory Res Pract Policy. (2020) 12:S180–2. 10.1037/tra0000852 32478539

[203] Bareket-Bojmel L Shahar G Abu-Kaf S Margalit M. Perceived social support, loneliness, and hope during the COVID-19 pandemic: testing a mediating model in the UK, USA, and Israel. Br J Clin Psychol. (2021) 60:133–48. 10.1111/bjc.12285 33624294 PMC8013849

[204] Ranieri V Sem Stoltenberg A Pizzo E Montaldo C Bizzi E Edwards S. COVID-19 welbeing study: a protocol examining perceived coercion and psychological well-being during the COVID-19 pandemic by means of an online survey, asynchronous virtual focus groups and individual interviews. BMJ Open. (2021) 11:e043418. 10.1136/bmjopen-2020-043418 33495259 PMC7839305

[205] Rosenfeld DL Balcetis E Bastian B Berkman ET Bosson JK Brannon TN. Psychological science in the wake of Covid-19: social, methodological, metascientific considerations. Perspect Psychol Sci. (2022) 17:311–33. 10.1177/1745691621999374 34597198 PMC8901450

[206] Vashdi DR, Chen, J, Bamberger, PA,. Buffering CoVID-Related Negative Emotional States Through Pre-Lockdown Team Interdependence Social Support. (2022). Available online at: https://obcovid19files.s3.amazonaws.com/vashdi.pdf

[207] Suzuki M Furihata R Konno C Kaneita Y Ohida T Uchiyama M. Stressful events and coping strategies associated with symptoms of depression: a Japanese general population survey. J Affect Disord. (2018) 238:482–8. 10.1016/j.jad.2018.06.024 29933216

[208] Hawton K van Heeringen K. Suicide. Lancet. (2009) 373:1372–81. 10.1016/S0140-6736(09)60372-X 19376453

[209] Tanaka T Okamoto S. Increase in suicide following an initial decline during the COVID-19 pandemic in Japan. Nat Hum Behav. (2021) 5:229–38. 10.1038/s41562-020-01042-z 33452498

[210] Hobfoll SE Vinokur AD Pierce PF Lewandowski-Romps L. The combined stress of family life, work, and war in air force men and women: a test of conservation of resources theory. Int J Stress Manage. (2012) 19:217–37. 10.1037/a0029247

[211] Gauthier GR Smith JA García C Garcia MA Thomas PA. Exacerbating inequalities: social networks, racial/ethnic disparities, and the COVID-19 pandemic in the United States. J Gerontol Ser B. (2020) 76:e88–e92. 10.1093/geronb/gbaa117 32756978 PMC7454830

[212] Thomas J Barbato M Verlinden M Gaspar C Moussa M Ghorayeb J. Psychosocial correlates of depression and anxiety in the united arab emirates during the COVID-19 pandemic. Front Psychiatry. (2020) 11:564172. 10.3389/fpsyt.2020.564172 33240122 PMC7683430

[213] Alonzi S La Torre A Silverstein MW. The psychological impact of preexisting mental and physical health conditions during the COVID-19 pandemic. Psychol Trauma Theory Res Pract Policy. (2020) 12:S236–8. 10.1037/tra0000840 32525380

[214] Van Lancker W Parolin Z. COVID-19, school closures, and child poverty: a social crisis in the making. Lancet Public Health. (2020) 5:e243–4. 10.1016/S2468-2667(20)30084-0 32275858 PMC7141480

[215] Chen S Westman M Hobfoll SE. The commerce and crossover of resources: resource conservation in the service of resilience. Stress Health. (2015) 31:95–105. 10.1002/smi.2574 25873421 PMC4564014

[216] Calhoun LG Tedeschi RG. Facilitating Posttraumatic Growth: A Clinician's Guide. New York, NY: Routledge (1999).

[217] Yi-Feng Chen N Crant JM Wang N Kou Y Qin Y Yu J. When there is a will there is a way: the role of proactive personality in combating COVID-19. J Appl Psychol. (2021) 106:199–213. 10.1037/apl0000865 33600195

[218] Cushing L Morello-Frosch R Wander M Pastor M. The haves, the have-nots, and the health of everyone: the relationship between social inequality and environmental quality. Annu Rev Public Health. (2015) 36:193–209. 10.1146/annurev-publhealth-031914-122646 25785890

[219] Neckerman KM Torche F. Inequality: causes and consequences. Ann Rev Sociol. (2007) 33:335–357. 10.1146/annurev.soc.33.040406.131755

[220] Fiske A Galasso I Eichinger J McLennan S Radhuber I Zimmermann B. The second pandemic: examining structural inequality through reverberations of COVID-19 in Europe. Soc Sci Med. (2022) 292:114634. 10.1016/j.socscimed.2021.114634 34883310 PMC8648175

[221] World Bank. Global Economic Prospects, 2020. Washington, DC: World Bank (2020). Available online at: http://hdl.handle.net/10986/33748

[222] Lustig N Arias O Rigolini J. Poverty Reduction and Economic Growth: a Two-way Casuality. Inter-American Development Bank, Sustainable Development Department (2002)

[223] WorldBank WB,. Poverty Overview. World Bank (2022). Retrieved from https://www.worldbank.org/en/topic/poverty/overview#1 (accessed May 19, 2022).

[224] Joffe A Redman D. Applying philosophy, logic, and rational argumentation to the severe acute respiratory syndrome Coronavirus-2 pandemic response. Preprints. (2021) 2021:2021050264. 10.20944/preprints202105.0264.v1

[225] Koh WC Alikhan MF Koh D Wong J. Containing COVID-19: implementation of early and moderately stringent social distancing measures can prevent the need for large-scale lockdowns. Ann Glob Health. (2020) 86:2969 10.5334/aogh.2969 32775219 PMC7394195

[226] Meyerowitz-Katz G Bhatt S Ratmann O Brauner JM Flaxman S Mishra S. Is the cure really worse than the disease? The health impacts of lockdowns during COVID-19. BMJ Glob Health. (2021) 6:e006653. 10.1136/bmjgh-2021-006653 34281914 PMC8292804

[227] Chin V Ioannidis JP Tanner MA Cripps S. Effect estimates of COVID-19 non-pharmaceutical interventions are non-robust and highly model-dependent. J Clin Epidemiol. (2021) 136:96–132. 10.1016/j.jclinepi.2021.03.014 33781862 PMC7997643

[228] Pak A Adegboye OA McBryde ES. Are we better-off? The benefits and costs of australian covid-19 lockdown. Front Public Health. (2021) 9:798478. 10.3389/fpubh.2021.798478 34926400 PMC8674450

[229] Arora AES Herrin J Riley C Roy B Kell K Coberley C. Population well-being measures help explain geographic disparities in life expectancy at the county level. Health Aff. (2016) 35:2075–82. 10.1377/hlthaff.2016.0715 27834249 PMC5150263

[230] Frijters P Clark AE Krekel C Layard R. A happy choice: wellbeing as the goal of government. Behav Public Policy. (2020) 4:126–65. 10.1017/bpp.2019.39

[231] Bartram D. Does inequality exacerbate status anxiety among higher earners? A longitudinal evaluation. Int J Compar Sociol. (2022) 2022:00207152221094815. 10.1177/00207152221094815

[232] Diener E Chan MY. Happy people live longer: subjective well-being contributes to health and longevity. Appl Psychol Health Well Being. (2011) 3:1–43. 10.1111/j.1758-0854.2010.01045.x

[233] Chida Y Steptoe A. Positive psychological well-being and mortality: a quantitative review of prospective observational studies. Psychosom Med. (2008) 70:741–56. 10.1097/PSY.0b013e31818105ba 18725425

[234] Bower M Buckle C Rugel E Donohoe-Bales A McGrath L Gournay K. ‘Trapped', ‘anxious' and ‘traumatised': COVID-19 intensified the impact of housing inequality on Australians' mental health. Int J Hous Policy. (2021) 32:1940686. 10.1080/19491247.2021.1940686

[235] Stantcheva S. Inequalities in the Times of a Pandemic (No. w29657). Cambridge, MA: National Bureau of Economic Research (2022). 10.3386/w29657

[236] Xiong J Lipsitz O Nasri F Lui LMW Gill H Phan L. Impact of COVID-19 pandemic on mental health in the general population: a systematic review. J Affect Disord. (2020) 277:55–64. 10.1016/j.jad.2020.08.001 32799105 PMC7413844

[237] McNeely CL Schintler LA Stabile B. Social determinants and COVID-19 disparities: differential pandemic effects and dynamics. World Med Health Policy. (2020) 12:206–17. 10.1002/wmh3.370 PMC7753789 33362940

[238] Yamey G McDade KK Brennan RJ Abubakar A Khan W. Preventing pandemics in the world's most vulnerable settings. BMJ. (2021) 375:n2897. 10.1136/bmj.n2897 34824126

[239] McCartney G Leyland A Walsh D Ruth D. Scaling COVID-19 against inequalities: should the policy response consistently match the mortality challenge? J Epidemiol Community Health. (2020) 75:315–20. 10.1136/jech-2020-214373 33144334 PMC7958082

[240] Clouston S Natale G Link BG. Socioeconomic inequalities in the spread of coronavirus-19 in the United States: a examination of the emergence of social inequalities. Soc Sci Med. (2021) 268:113554. 10.1016/j.socscimed.2020.113554 33308911 PMC7703549

[241] Gauvin L Barnett TA Dea C Doré I Drouin O Frohlich KL. Quarantots, quarankids, and quaranteens: how research can contribute to mitigating the deleterious impacts of the COVID-19 pandemic on health behaviours and social inequalities while achieving sustainable change. [Les tout-petits, enfants et ados de la quarantaine: contributions de la recherche à des changements durables pour mitiger les impacts délétères de la pandémie de COVID-19 sur les habitudes de vie et les inégalités sociales.] *Can J Public Health*. (2022) 113:53–60. 10.17269/s41997-021-00569-6 35089590 PMC8796597

[242] Ribeiro AL Alves Sousa NW Martins-Filho PR Carvalho VO. Social disparity in magnifying glass: the inequality among the vulnerable people during COVID-19 pandemic. Int J Clin Pract. (2021) 75:e13839. 10.1111/ijcp.13839 33202097 PMC7744851

[243] Barnard S Fryers P Fitzpatrick J Fox S Waller Z Baker A. Inequalities in excess premature mortality in England during the COVID-19 pandemic: a cross-sectional analysis of cumulative excess mortality by area deprivation and ethnicity. BMJ Open. (2021) 11:e052646. 10.1136/bmjopen-2021-052646 34949618 PMC8710653

[244] Blundell R Costa Dias M Joyce R Xu X. COVID-19 and inequalities. Fisc Stud. (2020) 41:219–319. 10.1111/1475-5890.12232 32836542 PMC7362053

[245] Cifuentes MP Rodriguez-Villamizar LA Rojas-Botero ML Alvarez-Moreno CA Fernández-Niño JA. Socioeconomic inequalities associated with mortality for COVID-19 in Colombia: a cohort nationwide study. J Epidemiol Community Health. (2021) 4:e216275. 10.1136/jech-2020-216275 33674459

[246] Liao TF De Maio F. Association of social and economic inequality with coronavirus disease 2019 incidence and mortality across US counties. JAMA Netw Open. (2021) 4:e2034578. 10.1001/jamanetworkopen.2020.34578 33471120 PMC7818127

[247] Perry BL Aronson B Pescosolido BA. Pandemic precarity: COVID-19 is exposing and exacerbating inequalities in the American heartland. Proc Natl Acad Sci USA. (2021) 118:e2020685118. 10.1073/pnas.2020685118 33547252 PMC7923675

[248] Watkinson RE Williams R Gillibrand S Sanders C Sutton M. Ethnic inequalities in COVID-19 vaccine uptake and comparison to seasonal influenza vaccine uptake in Greater Manchester, UK: a cohort study. PLoS Med. (2022) 19:e1003932. 10.1371/journal.pmed.1003932 35239661 PMC8893324

[249] González-Rábago Y Cabezas-Rodríguez A Martín U. Social inequalities in health determinants in spanish children during the COVID-19 lockdown. Int J Environ Res Public Health. (2021) 18:4087. 10.3390/ijerph18084087 33924441 PMC8069937

[250] Parker RF Figures EL Paddison CA Matheson JI Blane DN Ford JA. Inequalities in general practice remote consultations: a systematic review. BJGP Open. (2021) 5:40. 10.3399/BJGPO.2021.0040 33712502 PMC8278507

[251] Politi J Martín-Sánchez M Mercuriali L Borras-Bermejo B Lopez-Contreras J Vilella A. Epidemiological characteristics and outcomes of COVID-19 cases: mortality inequalities by socio-economic status, Barcelona, Spain, 24 February to 4 May 2020. Euro Surveill. (2021) 26:2001138. 10.2807/1560-7917.ES.2021.26.20.2001138 34018483 PMC8138960

[252] Reboucas P Falcão IR Barreto ML. Social inequalities and their impact on children's health: a current and global perspective. J Pediatr. (2022) 98 (Suppl. 1):S55–65. 10.1016/j.jped.2021.11.004 34951980 PMC9510930

[253] Jaspal R. Identity threat and coping among British South Asian gay men during the COVID-19 lockdown. Sex Cult. (2021) 25:1428–46. 10.1007/s12119-021-09817-w 33584091 PMC7871943

[254] Nemati S Saeedi E Abdi S Qandian A Kalhor E Moradi S. Decomposition of socioeconomic inequality in COVID-19 mortality in Iran: a retrospective cohort study. Health Soc Care Commu. (2021). 10.1111/hsc.13627 34738684 PMC8653285

[255] Sepulveda ER Brooker AS. Income inequality and COVID-19 mortality: Age-stratified analysis of 22 OECD countries. SSM Popul Health. (2021) 16:100904. 10.1016/j.ssmph.2021.100904 34584934 PMC8456048

[256] Bambra C Riordan R Ford J Matthews F. The COVID-19 pandemic and health inequalities. J epidemiol Community Health. (2020) 74:964–8. 10.1136/jech-2020-214401 32535550 PMC7298201

[257] Cerqua A Letta M. Local inequalities of the COVID-19 crisis. Reg Sci Urban Econ. (2022) 92:103752. 10.1016/j.regsciurbeco.2021.103752 34785828 PMC8585964

[258] Malmusi D Pasarín MI Marí-Dell'Olmo M Artazcoz L Diez E Tolosa S. Multi-level policy responses to tackle socioeconomic inequalities in the incidence of COVID-19 in a European urban area. Int J Equity Health. (2022) 21:28. 10.1186/s12939-022-01628-1 35183189 PMC8857870

[259] Tan AX Hinman JA Abdel Magid HS Nelson LM Odden MC. Association between income inequality and county-level COVID-19 cases and deaths in the US. JAMA Netw Open. (2021) 4:e218799. 10.1001/jamanetworkopen.2021.8799 33938935 PMC8094008

[260] Gao X Davillas A Jones AM. The Covid-19 pandemic and its impact on socioeconomic inequality in psychological distress in the United Kingdom: an update. Health Econ. (2022) 31:912–920. 10.1002/hec.4480 35170145

[261] Alicandro G Corsetti G Battaglini M Prati S Frova L. Education inequalities in overall mortality during the first wave of the COVID-19 pandemic in Italy. [Disuguaglianze per istruzione nella mortalità totale durante la prima ondata della pandemia di COVID-19 in Italia.] *Epidemiol Prev*. (2021) 45:463–9. 10.19191/ep21.6.122 35001594

[262] Stok FM Bal M Yerkes MA de Wit J. Social inequality and solidarity in times of COVID-19. Int J Environ Res Public Health. (2021) 18:6339. 10.3390/ijerph18126339 34208121 PMC8296166

[263] Jones N Baird S Abu Hamad B Bhutta ZA Oakley E Shah M. Compounding inequalities: adolescent psychosocial wellbeing and resilience among refugee and host communities in Jordan during the COVID-19 pandemic. PLoS ONE. (2022) 17:e0261773. 10.1371/journal.pone.0261773 35108293 PMC8809558

[264] Andrasfay T Goldman N. Association of the COVID-19 pandemic with estimated life expectancy by race/ethnicity in the United States, 2020. JAMA Netw Open. (2021) 4:e2114520. 10.1001/jamanetworkopen.2021.14520 34165582 PMC8226419

[265] Gundersen C Hake M Dewey A Engelhard E. Food insecurity during COVID-19. Appl Econ Perspect Policy. (2021) 43:153–61. 10.1002/aepp.13100 33042509 PMC7537061

[266] Laborde D Martin W Vos R. Poverty and Food Insecurity Could Grow Dramatically as COVID-19 Spreads. Washington, DC: International Food Policy Research Institute (IFPRI), (2020). 10.2499/p15738coll2.133762_02

[267] Niles MT Bertmann F Belarmino EH Wentworth T Biehl E Neff R. The early food insecurity impacts of COVID-19. Nutrients. (2020) 12:2096. 10.3390/nu12072096 32679788 PMC7400862

[268] Udmale P Pal I Szabo S Pramanik M Large A. Global food security in the context of COVID-19: a scenario-based exploratory analysis. Prog Disaster Sci. (2020) 7:100120. 10.1016/j.pdisas.2020.100120 34173442 PMC7374119

[269] Haelermans C Korthals R Jacobs M de Leeuw S Vermeulen S van Vugt L. Sharp increase in inequality in education in times of the COVID-19-pandemic. PLoS ONE. (2022) 17:e0261114. 10.1371/journal.pone.0261114 35108273 PMC8809564

[270] Andrew A Cattan S Costa Dias M Farquharson C Kraftman L Krutikova S. Inequalities in children's experiences of home learning during the COVID-19 lockdown in England. Fisc Stud. (2020) 41:653–683. 10.1111/1475-5890.12240 33362314 PMC7753283

[271] Katz VS Jordan AB Ognyanova K. Digital inequality, faculty communication, and remote learning experiences during the COVID-19 pandemic: A survey of U.S. undergraduates. PLoS ONE. (2021) 16:e0246641. 10.1371/journal.pone.0246641 33566832 PMC7875367

[272] Nguyen MH Hargittai E Marler W. Digital inequality in communication during a time of physical distancing: the case of COVID-19. Comput Hum Behav. (2021) 120:106717. 10.1016/j.chb.2021.106717 34751201 PMC8565917

[273] Zachreson C Martino E Tomko M Shearer FM Bentley R Geard N. Mapping home internet activity during COVID-19 lockdown to identify occupation related inequalities. Sci Rep. (2021) 11:21054. 10.1038/s41598-021-00553-7 34702880 PMC8548542

[274] Borrescio-Higa F Valenzuela P. Gender inequality and mental health during the covid-19 pandemic. Int J Public Health. (2021) 66:1604220. 10.3389/ijph.2021.1604220 34955701 PMC8698135

[275] Gibson B Schneider J Talamonti D Forshaw M. The impact of inequality on mental health outcomes during the COVID-19 pandemic: A systematic review. Can Psychol. (2021) 62:101–126. 10.1037/cap0000272

[276] Utzet M Bacigalupe A Navarro A. Occupational health, frontline workers and COVID-19 lockdown: new gender-related inequalities? J Epidemiol Community Health. (2022) 76.6:537–43. 10.1136/jech-2021-217692 35228295

[277] Yerkes MA André S Besamusca JW Kruyen PM Remery C van der Zwan R. 'Intelligent' lockdown, intelligent effects? Results from a survey on gender (in)equality in paid work, the division of childcare and household work, and quality of life among parents in the Netherlands during the Covid-19 lockdown. PLoS ONE. (2020) 15:e0242249. 10.1371/journal.pone.0242249 33253238 PMC7703961

[278] Fisher AN Ryan MK. Gender inequalities during COVID-19. Group Process Intergr Relat. (2021) 24:237–45. 10.1177/1368430220984248

[279] Nourazari S Davis SR Granovsky R Austin R Straff DJ Joseph JW. Decreased hospital admissions through emergency departments during the COVID-19 pandemic. Am J Emerge. (2021) 42:203–10. 10.1016/j.ajem.2020.11.029 33279331 PMC7676321

[280] Brzezinski M. The impact of past pandemics on economic and gender inequalities. Econ Hum Biol. (2021) 43:101039. 10.1016/j.ehb.2021.101039 34247056

[281] Christl M De Poli S Kucsera D Lorenz H. COVID-19 and (gender) inequality in income: the impact of discretionary policy measures in Austria. Swiss J Econ Stat. (2022) 158:4. 10.1186/s41937-022-00084-6 35155286 PMC8817162

[282] Dang HH Viet Nguyen C. Gender inequality during the COVID-19 pandemic: income, expenditure, savings, job loss. World Dev. (2021) 140:105296. 10.1016/j.worlddev.2020.105296 34548740 PMC8446715

[283] Martinez-Bravo M Sanz C. Inequality and psychological well-being in times of COVID-19: evidence from Spain. Series J Spanish Econ Assoc. (2021) 12:489–548. 10.1007/s13209-021-00255-3 34777626 PMC8576792

[284] Pitzalis M Span ò E. Stay home and be unfair: the amplification of inequalities among families with young children during COVID-19. Euro J Educ. (2021) 56:595–606. 10.1111/ejed.12481 34898741 PMC8646934

[285] Gorska AM Kulicka K Staniszewska Z Dobija D. Deepening inequalities: What did COVID-19 reveal about the gendered nature of academic work? Gend Work Organ. (2021) 18:12696. 10.1111/gwao.12696 34219993 PMC8239576

[286] Pinho-Gomes AC Peters S Thompson K Hockham C Ripullone K Woodward M. Where are the women? Gender inequalities in COVID-19 research authorship. BMJ Glob Health. (2020) 5:e002922. 10.1136/bmjgh-2020-002922 32527733 PMC7298677

[287] Quak E Girault G Thenint MA Weyts K Lequesne J Lasnon C. Author gender inequality in medical imaging journals and the COVID-19 pandemic. Radiology. (2021) 300:E301–7. 10.1148/radiol.2021204417 33724061 PMC7983071

[288] Guerrina R Borisch B Callahan LF Howick J Reginster JY Mobasheri A. Health and gender inequalities of the COVID-19 pandemic: adverse impacts on women's health, wealth and social welfare. Front Glob Womens Health. (2021) 2:670310. 10.3389/fgwh.2021.670310 34816222 PMC8593989

[289] Bellizzi S Nivoli A Lorettu L Ronzoni AR. Human rights during the COVID-19 pandemic: the issue of female genital mutilations. Public Health. (2020) 185:53–4. 10.1016/j.puhe.2020.05.037 32563099 PMC7247461

[290] Aburto JM Schöley J Kashnitsky I Zhang L Rahal C Missov TI. Quantifying impacts of the COVID-19 pandemic through life-expectancy losses: a population-level study of 29 countries. Int J Epidemiol. (2022) 51:63–74. 10.1093/ije/dyab207 34564730 PMC8500096

[291] Viner R Russell S Saulle R Croker H Stansfield C Packer J. School closures during social lockdown and mental health, health behaviors, and well-being among children and adolescents during the first COVID-19 wave: a systematic review. JAMA Pediatr. (2022) 176:400–9. 10.1001/jamapediatrics.2021.5840 35040870

[292] Bisht R Saharia R Sarma J. COVID-19 and the burden of ill-health: a double crisis of disruptions and inequalities. J Soc Econ Dev. (2020) 23.2:342–56. 10.1007/s40847-020-00117-x 34720476 PMC7747475

[293] Shur NF Johns D Kluzek S Peirce N. Physical inactivity and health inequality during coronavirus: a novel opportunity or total lockdown? BMJ Open Sport Exerc Med. (2020) 6:e000903. 10.1136/bmjsem-2020-000903 34422288 PMC8323465

[294] De Lorenzo A Cenname G Marchetti M Gualtieri P Dri M Carrano E. Social inequalities and nutritional disparities: the link between obesity and COVID-19. Eur Rev Med Pharmacol Sci. (2022) 26:320–39. Available at: https://www.europeanreview.org/wp/wp-content/uploads/320-339.pdf 35049011 10.26355/eurrev_202201_27784

[295] La Fauci G Montalti M Di Valerio Z Gori D Salomoni MG Salussolia A. Obesity and COVID-19 in children and adolescents: reciprocal detrimental influence—systematic literature review and meta-analysis. Int J Environ Res Public Health. (2022) 19:7603. 10.3390/ijerph19137603 35805260 PMC9266144

[296] Jaspal R Breakwell GM. Socio-economic inequalities in social network, loneliness and mental health during the COVID-19 pandemic. Int J Soc Psychiatry. (2022) 68:155–65. 10.1177/0020764020976694 33287610 PMC8793303

[297] Bendau A Viohl L Petzold MB Helbig J Reiche S Marek R. No party, no drugs? Use of stimulants, dissociative drugs, and GHB/GBL during the early COVID-19 pandemic. Int J Drug Policy. (2022) 102:103582. 10.1016/j.drugpo.2022.103582 35093679 PMC8730379

[298] Claes N Smeding A Carr é A. Mental health inequalities during COVID-19 outbreak: the role of financial insecurity and attentional control. Psychol Belg. (2021) 61:327–40. 10.5334/pb.1064 34824863 PMC8588930

[299] Fineberg NA Pellegrini L Wellsted D Hall N Corazza O Giorgetti V. Facing the “new normal”: how adjusting to the easing of COVID-19 lockdown restrictions exposes mental health inequalities. J Psychiatr Res. (2021) 141:276–86. 10.1016/j.jpsychires.2021.07.001 34271458 PMC7611491

[300] Gagne MH Piché G Clément MÈ Villatte A. Families in confinement: a pre–post COVID-19 study. Couple Fam Psychol Res Pract. (2021) 10:260–70. 10.1037/cfp0000179

[301] Gagne T Nandi A Schoon I. Time trend analysis of social inequalities in psychological distress among young adults before and during the pandemic: evidence from the uk household longitudinal study COVID-19 waves. J Epidemiol Community Health. (2021) 76:421–7. 10.1136/jech-2021-217266 34716130 PMC8561821

[302] Sudo N. The positive and negative effects of the COVID-19 pandemic on subjective well-being and changes in social inequality: evidence from prefectures in Japan. SSM Popul Health. (2022) 17:101029. 10.1016/j.ssmph.2022.101029 35079619 PMC8776341

[303] Esseau-Thomas C Galarraga O Khalifa S. Epidemics, pandemics and income inequality. Health Econ Rev. (2022) 12:7. 10.1186/s13561-022-00355-1 35043257 PMC8765494

[304] Bonacini L Gallo G Scicchitano S. Working from home and income inequality: risks of a 'new normal' with COVID-19. J Popul Econ. (2020) 34.1:1–58. 10.1007/s00148-020-00800-7 32952308 PMC7486597

[305] Delaporte I Escobar J Peña W. The distributional consequences of social distancing on poverty and labour income inequality in Latin America and the Caribbean. J Popul Econ. (2021) 1–59. 10.1007/s00148-021-00854-1 34334958 PMC8316545

[306] Palomino JC Rodríguez JG Sebastian R. Wage inequality and poverty effects of lockdown and social distancing in Europe. Euro Econ Rev. (2020) 129:103564. 10.1016/j.euroecorev.2020.103564 32836323 PMC7417923

[307] Perugini C Vladisavljevi ć M. Social stability challenged by Covid-19: pandemics, inequality and policy responses. J Policy Model. (2021) 43:146–60. 10.1016/j.jpolmod.2020.10.004 33311816 PMC7718105

[308] Shen J Shum WY Cheong TS Wang L. COVID-19 and regional income inequality in China. Front Public Health. (2021) 9:687152. 10.3389/fpubh.2021.687152 34046393 PMC8144473

[309] Deaton A. COVID-19 and global income inequality. LSE Public Policy Rev. (2021) 1:1. 10.31389/lseppr.26 34308354 PMC8301493

[310] Bacher-Hicks A Goodman J Mulhern C. Inequality in household adaptation to schooling shocks: COVID-induced online learning engagement in real time. J Public Econ. (2021) 193:104345. 10.1016/j.jpubeco.2020.104345 34629567 PMC8486492

[311] Devkota KR. Inequalities reinforced through online and distance education in the age of COVID-19: the case of higher education in Nepal. Int Rev Educ. (2021) 67:145–65. 10.1007/s11159-021-09886-x 33678863 PMC7925807

[312] Grewenig E Lergetporer P Werner K Woessmann L Zierow L. COVID-19 and educational inequality: how school closures affect low- and high-achieving students. Eur Econ Rev. (2021) 140:103920. 10.1016/j.euroecorev.2021.103920 34602646 PMC8474988

[313] Brooks SK Webster RK Smith LE Woodland L Wessely S Greenberg N. The psychological impact of quarantine and how to reduce it: rapid review of the evidence. Lancet. (2020) 395:912–20. 10.1016/S0140-6736(20)30460-8 32112714 PMC7158942

[314] Holt-Lunstad J Smith TB. Social relationships and mortality. Soc Person Psychol Compass. (2012) 6:41–53. 10.1111/j.1751-9004.2011.00406.x

[315] Holt-Lunstad J Smith TB Layton JB. Social relationships and mortality risk: a meta-analytic review. PLOS Med. (2010) 7:e1000316. 10.1371/journal.pmed.1000316 20668659 PMC2910600

[316] Czeisler MÉ Marynak K Clarke KE Salah Z Shakya I Thierry JM. Delay or avoidance of medical care because of COVID-19–related concerns—United States, June 2020. Morb Mortal Wkly Rep. (2020) 69:1250. 10.15585/mmwr.mm6936a4 32915166 PMC7499838

[317] Imlach F McKinlay E Kennedy J Pledger M Middleton L Cumming J. Seeking healthcare during lockdown: Challenges, opportunities and lessons for the future. Int J Health Policy Manag. (2021). 10.34172/ijhpm.2021.26. [Epub ahead of print]. 33906337 PMC9808356

[318] Lange SJ Ritchey MD Goodman AB Dias T Twentyman E Fuld J. Potential indirect effects of the COVID-19 pandemic on use of emergency departments for acute life-threatening conditions — United States, January–May 2020. Am J Transplant. (2020) 20:2612–7. 10.1111/ajt.16239 32862556 PMC9800659

[319] Saeki H Shirabe K Miyazaki T Ogawa T Makita F Shitara Y. Decreased numbers of gastric, colorectal, lung, and breast cancer surgeries performed in 17 cancer-designated hospitals in Gunma prefecture of Japan during the COVID-19 pandemic. Surgery Today. (2022) 15:1–7. 10.1007/s00595-022-02501-y 35426582 PMC9010936

[320] Collaborative C. Elective surgery cancellations due to the COVID-19 pandemic: global predictive modelling to inform surgical recovery plans. Br J Surg. (2020) 107:1440–9. 10.1002/bjs.11746 32395848 PMC7272903

[321] Arnault L Jusot F Renaud T. Economic vulnerability and unmet healthcare needs among the population aged 50 + years during the COVID-19 pandemic in Europe. Eur J Ageing. (2021) 5:1–15. 10.1007/s10433-021-00645-3 34512226 PMC8418894

[322] Ferreira F,. Inequality covid-19 – IMF FandD. International Monetary Fund – Homepage (2022). Retrieved from https://www.imf.org/external/pubs/ft/fandd/2021/06/inequality-and-covid-19-ferreira.htm#:~:text=The%20severe%20impact%20of%20the,extreme%3A%20the%20wealth%20of%20billionaires (accessed May 19, 2021).

[323] Wikipedia. Economic Inequality. Wikipedia (2022). Retrieved from https://en.wikipedia.org/wiki/Economic_inequality (accessed May 19, 2022).

[324] Yonzan N, Laknerdaniel, C, Mahler, G,. Is Covid-19 Increasing Global Inequality? World Bank Blogs (2022). Retrieved from https://blogs.worldbank.org/opendata/covid-19-increasing-global-inequality (accessed May 19, 2021).

[325] Berkhout E Galasso N Lawson M Rivero Morales PA Taneja A Vázquez Pimentel DA. The Inequality Virus: Bringing Together a World Torn Apart by Coronavirus Through a Fair, Just and Sustainable Economy. Oxford: Oxfam (2021) 10.21201/2021.6409

[326] Buheji M da Costa Cunha K Beka G Mavric B De Souza Y da Costa Silva SS. The extent of covid-19 pandemic socio-economic impact on global poverty. A global integrative multidisciplinary review. Am J Econ. (2020) 10:213–24. 10.5923/j.economics.20201004.0222499009

[327] Sanchez-Paramo C, Hill, R, Mahler, D, Narayan, A, Yonzar, N,. Covid-19 Leaves a Legacy of Rising Poverty Widening Inequality. World Bank Blogs (2022). Retrieved from https://blogs.worldbank.org/developmenttalk/covid-19-leaves-legacy-rising-poverty-and-widening-inequality (accessed May 19, 2021).

[328] Borkowski A Ortiz Correa JS Bundy DA Burbano C Hayashi C Lloyd-Evans E. COVID-19: Missing More than a Classroom. The Impact of School Closures on Children's Nutrition. Innocenti Working Paper 2021-01. UNICEF (2021).

[329] Blundell R Cribb J McNally S Warwick R Xu X. Inequalities in Education, Skills, and Incomes in the UK: The Implications of the COVID-19 Pandemic. Institute for Fiscal Studies (2021).

[330] Dorn E Hancock B Sarakatsannis J Viruleg E. COVID-19 and Student Learning in the United States: The Hurt Could Last a Lifetime. McKinsey and Company (2020).

[331] Engzell P Frey A Verhagen MD. Learning loss due to school closures during the COVID-19 pandemic. Proc Nat Acad Sci USA. (2021) 118:e2022376118. 10.1073/pnas.2022376118 33827987 PMC8092566

[332] Cantillon B Chzhen Y Handa S Nolan B. Children of Austerity: Impact of the Great Recession on Child Poverty in Rich Countries. Oxford: Oxford University Press (2017). 10.1093/oso/9780198797968.001.0001

[333] Prowse R Sherratt F Abizaid A Gabrys RL Hellemans KGC Patterson ZR. Coping with the COVID-19 pandemic: examining gender differences in stress and mental health among university students. Front Psychiatry. (2021) 12:650759. 10.3389/fpsyt.2021.650759 33897499 PMC8058407

[334] Malhi P Bharti B Sidhu M. Stress and parenting during the COVID-19 pandemic: psychosocial impact on children. Indian J Pediatr. (2021) 88:481. 10.1007/s12098-021-03665-0 33471315 PMC7815967

[335] Debowska A Horeczy B Boduszek D Dolinski D. A repeated cross-sectional survey assessing university students' stress, depression, anxiety, and suicidality in the early stages of the COVID-19 pandemic in Poland. Psychol Med. (2020) 2:1–4. 10.1017/S003329172000392X 33004087 PMC7556906

[336] Benassi E Vallone M Camia M Scorza M. Women during the covid- 19 lockdown: more anxiety symptoms in women with children than without children and role of the resilience. Mediterranean Clin Psychol. (2020) 8:1–19. 10.6092/2282-1619/mjcp-2559

[337] Fushimi M. The importance of studying the increase in suicides and gender differences during the COVID-19 pandemic. QJM. (2021) 115:57–8. 10.1093/qjmed/hcab130 33964169 PMC8135995

[338] Iob E Steptoe A Fancourt D. Abuse, self-harm and suicidal ideation in the UK during the COVID-19 pandemic. Br J Psychiatry. (2020) 217:543–6. 10.1192/bjp.2020.130 32654678 PMC7360935

[339] Kilius E Abbas NH McKinnon L Samson DR. Pandemic nightmares: COVID-19 lockdown associated with increased aggression in female university students' dreams. Front Psychol. (2021) 12:644636. 10.3389/fpsyg.2021.644636 33746860 PMC7973031

[340] United Nations. From Insights to Action: Gender Equality in the Wake of COVID-19. United Nations Entity for Gender Equality and the Empowerment of Women. New York, NY: UN Women (2020).

[341] Mittal S Singh T. Gender-based violence during COVID-19 pandemic: a mini-review. Front Glob Womens Health. (2020) 1:4. 10.3389/fgwh.2020.00004 34816149 PMC8594031

[342] Davenport MH Meyer S Meah VL Strynadka MC Khurana R. Moms are not ok: COVID-19 and maternal mental health. Front Glob Womens Health. (2020) 1:1. 10.3389/fgwh.2020.00001 34816146 PMC8593957

[343] Malisch JL Harris BN Sherrer SM Lewis KA Shepherd SL McCarthy PC. In the wake of COVID-19, academia needs new solutions to ensure gender equity. Proc Natl Acad Sci USA. (2020) 117:15378–81. 10.1073/pnas.2010636117 32554503 PMC7354923

[344] Collins C Landivar LC Ruppanner L Scarborough WJ. COVID-19 and the gender gap in work hours. Gend Work Organ. (2021) 28:101–12. 10.1111/gwao.12506 32837019 PMC7361447

[345] Monroe K Ozyurt S Wrigley T Alexander A. Gender equality in academia: bad news from the trenches, and some possible solutions. Perspect Polit. (2008) 6:215–33. 10.1017/S1537592708080572

[346] Woitowich NC Jain S Arora VM Joffe H. COVID-19 threatens progress toward gender equity within academic medicine. Acad Med. (2021) 96:813– 10.1097/ACM.0000000000003782 33003040 PMC7543905

[347] Cui R Ding H Zhu F. Gender inequality in research productivity during the COVID-19 pandemic. Manufact Serv Operat Manage. (2022) 24:707–26. 10.1287/msom.2021.099135529705

[348] Zimmer K,. Gender Gap in Research Output Widens During Pandemic. The Scientist (2020). Available online at: https://www.the-scientist.com/news-opinion/gender-gap-in-research-outputwidens-during-pandemic-67665 (accessed August 8, 2022).

[349] Ginette A Antra B Sara DE Sophie H Julia S Clare W. Spotlight on gender, COVID-19 and the SDGs: will the pandemic derail hardwon progress on gender equality? Spotlight on the SDGs. New York, NY: UN Women (2020). Available at: http://eprints.lse.ac.uk/id/eprint/105826

[350] Loeb TB Ebor MT Smith AM Chin D Novacek DM Hampton-Anderson JN. How mental health professionals can address disparities in the context of the COVID-19 pandemic. Traumatology. (2021) 27:60–9. 10.1037/trm0000292 34025223 PMC8132617

[351] Ribeiro WS Bauer A Andrade MCR York-Smith M Pan PM Pingani L. Income inequality and mental illness-related morbidity and resilience: a systematic review and meta-analysis. Lancet Psychiatry. (2017) 4:554–62. 10.1016/S2215-0366(17)30159-1 28552501

[352] Marmot MG Shipley MJ. Do socioeconomic differences in mortality persist after retirement? 25 Year follow up of civil servants from the first Whitehall study. BMJ. (1996) 313:1177–80. 8916748 10.1136/bmj.313.7066.1177 PMC2352486

[353] Sapolsky RM. Social status and health in humans and other animals. Ann Rev Anthropol. (2004) 33:393–418. 10.1146/annurev.anthro.33.070203.144000

[354] Fleischmann M Xue B Head J. Mental health before and after retirement—assessing the relevance of psychosocial working conditions: the whitehall ii prospective study of british civil servants. J Gerontol Ser B. (2019) 75:403–13. 10.1093/geronb/gbz042 31100154 PMC7392102

[355] Coccia M. How a good governance of institutions can reduce poverty and inequality in society? In: Faghih N, Samadi HA, editors. Legal-Economic Institutions, Entrepreneurship, and Management: Perspectives on the Dynamics of Institutional Change from Emerging Markets. Springer International Publishing (2021). 10.1007/978-3-030-60978-8_4

[356] Coleman DM Perrone EE Dombrowski J Dossett LA Sears ED Sandhu G. Overcoming COVID-19: strategies to mitigate the perpetuated gender achievement gap. Ann Surg. (2022) 275:435–7. 10.1097/SLA.0000000000005149 34387196 PMC8820744

[357] Seery E. Responding With Equality: The Case for Combating Extreme Inequality to Tackle Crises, Strengthen Democracy and Foster a Fairer Future in the Wake of the Coronavirus Pandemic. Oxfam (2021). 10.21201/2021.8281

[358] Erickson D Nancy A. (2011). Partnerships among community development, public health, and health care could improve the well-being of low-income people. Health Aff. 30:2056–63. 10.1377/hlthaff.2011.0896 22068396

[359] Eden L Wagstaff MF. Evidence-based policymaking and the wicked problem of SDG 5 gender equality. J Int Bus Policy. (2021) 4:28–57. 10.1057/s42214-020-00054-w

[360] Ioannidis JPA. The end of the COVID-19 pandemic. Euro J Clin Invest. (2022) 52:e13782. 10.1111/eci.13782 35342941 PMC9111437

[361] Inglesby TV Nuzzo JB O'Toole T Henderson DA. Disease mitigation measures in the control of pandemic influenza. Biosec Bioterror Biodefense Strat Pract Sci. (2006) 4:366–75. 10.1089/bsp.2006.4.366 17238820

[362] Redman D. An emergency management doctrine and philosophy: the five dimensions. Preprints. (2021) 2021:2021020367. 10.20944/preprints202102.0367.v1

[363] Redman D,. Canada's Deadly Response to COVID-19. Frontier Center for Public Policy. Policy Series No. 237. (2021). Available online at: https://fcpp.org/wp-content/uploads/FCPS237_CDADeadlyResponse_JL1621_F2.pdf

[364] Redman D,. A Recovery Plan. Canada's post-pandemic COVID-19. Frontier Center for Public Policy Briefing Note (2022). Available online at: https://fcpp.org/wp-content/uploads/BriefingNote-COVID_Recovery_PlanFB0922.pdf

[365] Zweig SA Zapf AJ Beyrer C Guha-Sapir D Haar RJ. Ensuring rights while protecting health: the importance of using a human rights approach in implementing public health responses to COVID-19. Health Hum Rights. (2021) 23:173. 34966234 PMC8694292

[366] Focacci CN Lam PH Bai Y. Choosing the right COVID-19 indicator: crude mortality, case fatality, and infection fatality rates influence policy preferences, behaviour, and understanding. Hum Soc Sci Commun. (2022) 9:19. 10.1057/s41599-021-01032-0

[367] Abbasi K. Covid-19: politicisation, “corruption,” and suppression of science. BMJ. (2020) 371:m4425. 10.1136/bmj.m4425 33187972

[368] Bavel JJV Baicker K Boggio PS Capraro V Cichocka A Cikara M. Using social and behavioural science to support COVID-19 pandemic response. Nat Hum Behav. (2020). 32355299 10.1038/s41562-020-0884-z

[369] Escandón K Rasmussen AL Bogoch II Murray EJ Escandón K Popescu SV. COVID-19 false dichotomies and a comprehensive review of the evidence regarding public health, COVID-19 symptomatology, SARS-CoV-2 transmission, mask wearing, and reinfection. BMC Infect Dis. (2021) 21:710. 10.1186/s12879-021-06357-4 34315427 PMC8314268

[370] Gupta KU, Sevimli, S, Arawi, T, Puentes, LV, Marlon, P,. Ethical Values Principles for Healing Society in Light of the COVID-19 Crisis. (2021). Available online at: https://www.eubios.info/yahoo_site_admin/assets/docs/WeCopeStatementHealingValues.20160152.pdf

[371] Rubin O Errett NA Upshur R Baekkeskov E. The challenges facing evidence-based decision making in the initial response to COVID-19. Scand J Public Health. (2021) 49:790–6. 10.1177/1403494821997227 33685289

[372] Joffe AR. What about the COVID-19 response? Evidence: risk, lockdowns, vaccine mandates. Health Ethics Today. (2022) 29:8–15. Available at: https://www.ualberta.ca/john-dossetor-health-ethics-centre/media-library/health-ethicstoday/health-ethics-today-volume29-1-february2022.pdf

[373] Freyhofer S Ziegler N de Jong EM and Schippers MC. Depression and anxiety in times of covid-19: how coping strategies and loneliness relate to mental health outcomes and academic performance. Front Psychol. (2021) 12:682684. 10.3389/fpsyg.2021.682684 34759855 PMC8572913

[374] Bateman TS Crant JM. The proactive component of organizational behavior: a measure and correlates. J Organ Behav. (1993) 14:1031118.

[375] Crant JM Hu J Jiang K. Proactive personality: a twenty-year review. Proact Work. (2016) 1:211–43. 10.4324/9781315797113-17

[376] Kalia SK,. Closing the Global Gender Inequality Gap Will Take 135 Years, New Report Finds. The Swaddle (2022). Retrieved from https://theswaddle.com/closing-the-global-gender-inequality-gap-will-take-135-years-new-report-finds/ (accessed May 19, 2021).

[377] World Economic Forum. Global Gender Gap Report 2021. World Economic Forum (2021). Available online at: https://www.weforum.org/reports/global-gender-gap-report-2021 (accessed April 21, 2022).

[378] Saul JR. Voltaire's Bastards: The Dictatorship of Reason in the West. New York, NY: Simon and Schuster (2013).

[379] Conti P. Trauma: the Invisible Epidemic: How Trauma Works and How We Can Heal From it. Denver, CO: Sounds True (2021).

[380] Shapiro R Brown LS. Eye movement desensitization and reprocessing therapy and related treatments for trauma: an innovative, integrative trauma treatment. Pract Innov. (2019) 4:139. 10.1037/pri0000092

[381] Grand S. The Reproduction of Evil: A Clinical and Cultural Perspective. New York, NY: Routledge (2013). 10.4324/9780203767245

[382] Wimberger L. Neurosculpting: A Whole-Brain Approach to Heal Trauma, Rewrite Limiting Beliefs, Find Wholeness. Denver, CO: Sounds True (2015).

[383] D'Antoni F Feruglio S Matiz A Cantone D Crescentini C. Mindfulness meditation leads to increased dispositional mindfulness and interoceptive awareness linked to a reduced dissociative tendency. J Trauma Dissociat. (2022) 23:8–23. 10.1080/15299732.2021.1934935 34076566

[384] Gurda K. Emerging trauma therapies: Critical analysis and discussion of three novel approaches. J Aggress Maltreat Trauma. (2015) 24:773–93. 10.1080/10926771.2015.1062445

[385] Schippers MC Ziegler N. Life crafting as a way to find purpose and meaning in life. Front Psychol. (2019) 10:2778. 10.3389/fpsyg.2019.02778 31920827 PMC6923189

[386] Dekker I De Jong EM Schippers MC De Bruijn-Smolders M Alexiou A Giesbers B. Optimizing students' mental health and academic performance: ai-enhanced life crafting. Front Psychol. (2020) 11:1063. 10.3389/fpsyg.2020.01063 32581935 PMC7286028

[387] Hoermann S McCabe KL Milne DN Calvo RA. Application of synchronous text-based dialogue systems in mental health interventions: systematic review. J Med Internet Res. (2017) 19:e7023. 10.2196/jmir.7023 28784594 PMC5595406

[388] Kleiman EM Adams LM Kashdan TB Riskind JH. Gratitude and grit indirectly reduce risk of suicidal ideations by enhancing meaning in life: evidence for a mediated moderation model. J Res Person. (2013) 47:539–46. 10.1016/j.jrp.2013.04.007

[389] Wood AM Froh JJ Geraghty AWA. Gratitude and well-being: a review and theoretical integration. Clin Psychol Rev. (2010) 30:890–905. 10.1016/j.cpr.2010.03.005 20451313

[390] Liao KYH Weng CY. Gratefulness and subjective well-being: social connectedness and presence of meaning as mediators. J Counsel Psychol. (2018) 65:383–93. 10.1037/cou0000271 29672087

[391] Schippers MC Scheepers AWA Peterson JB. A scalable goal-setting intervention closes both the gender and ethnic minority achievement gap. Palgrave Commun. (2015) 1:15014. 10.1057/palcomms.2015.14

[392] Easterbrook MJ Hadden IR. Tackling educational inequalities with social psychology: identities, contexts, and interventions. Soc Issues Policy Rev. (2021) 15:180–236. 10.1111/sipr.12070

[393] Kaluza AJ van Dick R. Telework at times of a pandemic: the role of voluntariness in the perception of disadvantages of telework. Curr Psychol. (2022) 1:1–12. 10.1007/s12144-022-03047-5 35382038 PMC8970638

[394] Afsahi A Beausoleil E Dean R Ercan SA Gagnon J.-P. Democracy in a global emergency: five lessons from the COVID-19 pandemic. Democrat Theory. (2020) 7:v–xix. 10.3167/dt.2020.070201

[395] Dostal JM. Germany's corona crisis: the authoritarian turn in public policy and the rise of the biosecurity state (2020-2022). J Korean German Assoc Soc Sci. (2022) 32:143–88. 10.19032/zkdgs.2022.03.32.1.143

[396] Stoker G Evans M. Saving Democracy. London: Bloomsbury Publishing (2022).

[397] Newman A Freilekhman I. A case for regulated industrial democracy post-Covid-19. N Zeal J Employ Relat. (2020) 45:70–6. 10.24135/nzjer.v45i2.29

[398] Elm JP Sarel R. Partially right means generally wrong: why some Covid-19 mitigation strategies keep on failing. SSRN. (2021) 3775020. 10.2139/ssrn.3775020

[399] Hirt J Janiaud P Hemkens LG. Randomized trials on non-pharmaceutical interventions for COVID-19: a scoping review. BMJ Evid Bas Med. (2022) 2021:111825. 10.1136/bmjebm-2021-111825 35086864 PMC8804305

[400] Janiaud P Hemkens LG Ioannidis JPA. Challenges and lessons learned from COVID-19 trials: should we be doing clinical trials differently? Can J Cardiol. (2021) 37:1353–64. 10.1016/j.cjca.2021.05.009 34077789 PMC8164884

[401] Cristea IA Naudet F Ioannidis JPA. Preserving equipoise and performing randomised trials for COVID-19 social distancing interventions. Epidemiol Psychiatr Sci. (2020) 29:e184. 10.1017/S2045796020000992 33109299 PMC7674786

[402] Marmot M Allen J. COVID-19: exposing and amplifying inequalities. J Epidemiol Community Health. (2020) 74:681–2. 10.1136/jech-2020-214720 32669357 PMC7577092

[403] Whitehead M Taylor-Robinson D Barr B. Poverty, health, and covid-19. BMJ. (2021) 372:n376. 10.1136/bmj.n376 33579719

